# Novel Selenoureas Based on *Cinchona* Alkaloid Skeleton: Synthesis and Catalytic Investigations

**DOI:** 10.3390/ma14030600

**Published:** 2021-01-28

**Authors:** Mariola Zielińska-Błajet, Joanna Najdek

**Affiliations:** 1 Faculty of Chemistry, Wrocław University of Science and Technology, Wybrzeże Wyspiańskiego 27, 50-370 Wrocław, Poland; 2 Institute of Chemistry and Biochemistry, Freie Universität Berlin, Takustr. 3, 14195 Berlin, Germany; joanna.najdek@hotmail.com

**Keywords:** *Cinchona* alkaloids, selenoureas, isoselenocyanates, organocatalysis, Michael addition, asymmetric synthesis

## Abstract

An efficient approach to the synthesis of chiral selenoureas consisting of *Cinchona* alkaloid scaffolds was described. The new selenoureas were assessed as bifunctional organocatalysts in the asymmetric Michael addition reactions under mild conditions. The best results were obtained for selenoureas bearing the 4-fluorophenyl group. These catalysts promoted the reactions with enantioselectivities of up to 96% ee. Additionally, the catalytic performance of the thiourea and selenourea counterpart was compared.

## 1. Introduction

In recent years, growing attention has been focused on the synthesis and applications of selenourea derivatives. The structures of selenoureas are closely related to those of analogs of oxygen and sulfur compounds [1,2,3], but the presence of larger, more polarizable selenium results in a change of significant properties. They possess various biological properties, such as antioxidant [4,5], antibacterial and antifungal [6], antileishmanial [7], pesticidal [8], and anti-urease activity [9]. Selenoureas also demonstrate enzyme inhibition, free-radical scavenging, and anticancer activity [10,11]. These compounds are very useful starting materials for the synthesis of selenium-containing heterocycles [12,13] and are used for anion binding and recognition [14,15]. To the best of our knowledge, only a few examples of chiral selenoureas have been reported in the literature [16,17]. Currently, chiral bis-selenourea is used as a strong hydrogen-bonding donor for highly efficient chiral recognition of a diverse range of tertiary alcohols [18]. Still, little is known about the application of selenoureas as hydrogen-bond donors in organocatalysis. Bolm and co-workers were the first to apply a selenourea derivative as a chiral catalyst in the asymmetric Michael addition of α-nitrocyclohexane to aryl nitroalkenes [19].

Chiral bifunctional organocatalysts, which incorporate a thiourea group as H-bond donors with Lewis base, are commonly used in asymmetric catalysis. Both parts of the catalysts simultaneously activate the electrophile and nucleophile leading to the high stereocontrol of the reaction [20,21]. Nowadays, thioureas are widely recognized as highly useful and powerful organocatalysts that promote a diverse range of reactions with excellent enantioselectivity [22,23,24]. *Cinchona* alkaloids with an exceptionally good chiral backbone have been used for the synthesis of many successful catalysts [25]. Inspired by the improved catalytic activity of the thioureas in comparison to ureas, we have taken interest in the modification of the “privileged” *Cinchona* alkaloid scaffolds by substituting the thiourea with a selenourea moiety.

Here, we report the approach to the efficient synthesis of a series of novel *Cinchona* alkaloid-derived selenoureas, and we also demonstrate their organocatalytic potential in asymmetric Michael additions.

## 2. Results and Discussion

### 2.1. Preparation of Compounds

*Cinchona* alkaloid-based selenoureas were prepared by coupling reactions of 9-*epi*-aminoalkaloids with aryl isoselenocyanates. Although there are some methods in the literature for the preparation of selenoureas [4,10,26], the best procedure still turns out to be the reaction of isoselenocyanates with amines. The crucial step is the preparation of isoselenocyanates, which, unlike their sulfur analogs, are not commercially available. Several strategies towards their synthesis have been reported [27,28,29]. The oxidation of corresponding isocyanides with elemental black selenium in a suitable solvent seems to be the most reliable method [13,16,30,31].

**Scheme 1:**
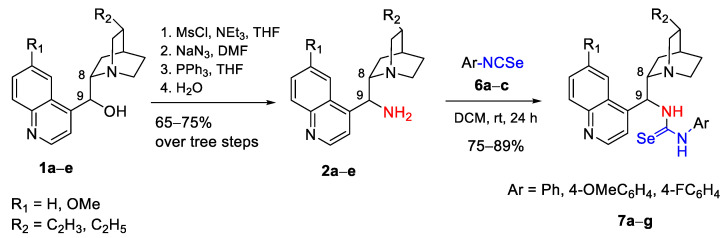
General synthetic route for compounds **7a**–**g**.

The first coupling partner for the synthesis of desired selenoureas was *Cinchona* alkaloid-based amines **2a**–**e**, which were prepared in good yields (65–75%) from naturally occurring *Cinchona* alkaloids, namely quinine (QN), cinchonidine (CD), quinidine (QD), and the respective dihydro derivatives (dihydroquinine (DHQN)/dihydroquinidine (DHQD)) in a three-step sequence via mesylation [32], azidation with NaN_3_ in warm DMF followed by S_N_2 displacement [33], and then treatment with triphenylphosphine (Staudinger reduction) to amine (Scheme 1) [34].

**Scheme 2:**
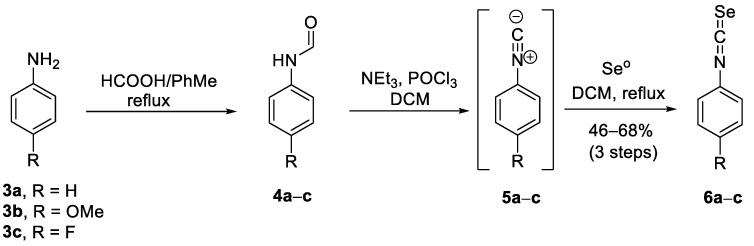
Synthesis of aryl isoselenocyanates **6a**–**c**.

Non-commercial aryl isoselenocyanates **6a**–**c**, the second coupling partner, were synthesized in good yields (46–68%) in a three-step protocol (Scheme 2) that involved *N*-formylation of the commercially available aryl amines **3a**–**c** with formic acid in toluene, conversion of the formamides **4a**–**c** into isocyanides **5a**–**c** upon dehydration with POCl_3_ in the presence of NEt_3_ in dichloromethane (DCM) [35]. The last step included the reaction of the isocyanides with black selenium in DCM at 45 °C in darkness.

**Figure 1:**
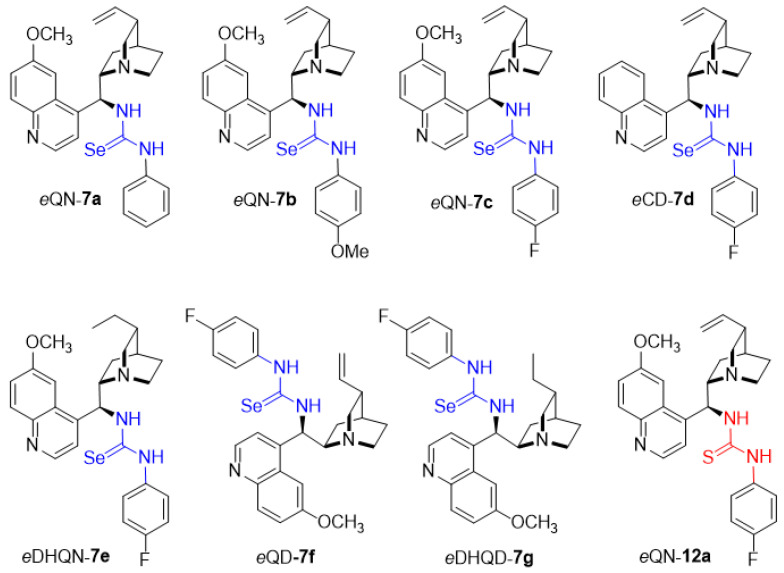
Structures of the *Cinchona*-based selenoureas.

The coupling of the aryl isoselenocyanates **6a**–**c** with 9-*epi*-aminoalkaloids **2a**–**e** in DCM at 45 °C in the darkness resulted in the formation of the desired selenoureas **7a**–**g** in high yields (75–89%) (Scheme 1), (Figure 1). All new compounds were fully characterized by spectroscopic methods (IR, ^1^H, and ^13^C NMR, HRMS). Copies of ^1^H, ^13^C NMR and IR spectra are presented in Supplementary Materials.

**Scheme 3:**
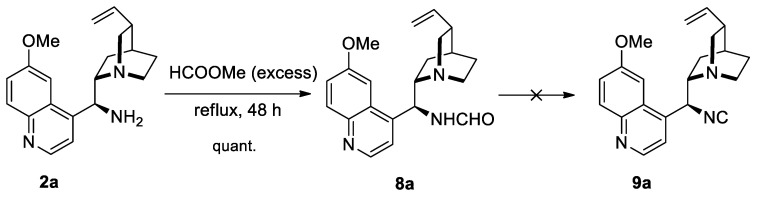
Unsuccessful attempts to prepare isocyanide **9a**.

In an alternative approach, we intended to introduce the isoselenocyanate moiety in the *Cinchona* alkaloid skeleton. 9*S*-Amino-deoxyquinine **2a** was chosen for the transformation into isoselenocyanate. Amine **2a** was converted into 9*S*-formylamino-deoxyquinine **8a** by treatment with the excess of methyl formate in quantitative yield (Scheme 3). The spectroscopic data of compound **8a** can be found in Supplementary Materials, Figures S15 and S16.

Surprisingly, dehydration of formamide **8a** using the previously applied POCl_3_/NEt_3_ system did not provide the desired isocyanide, and the amine **2a** was fully recovered. Due to this unsatisfying result, other known methods to convert formamide **8a** to isocyanide **9a** were tested. Hence, the procedures using the triphosgene/NEt_3_/DCM and PPh_3_/CCl_4_/NEt_3_ system as well as Vilsmeier and Burgess reagents turned out to be ineffective [17,30,36].

**Scheme 4:**
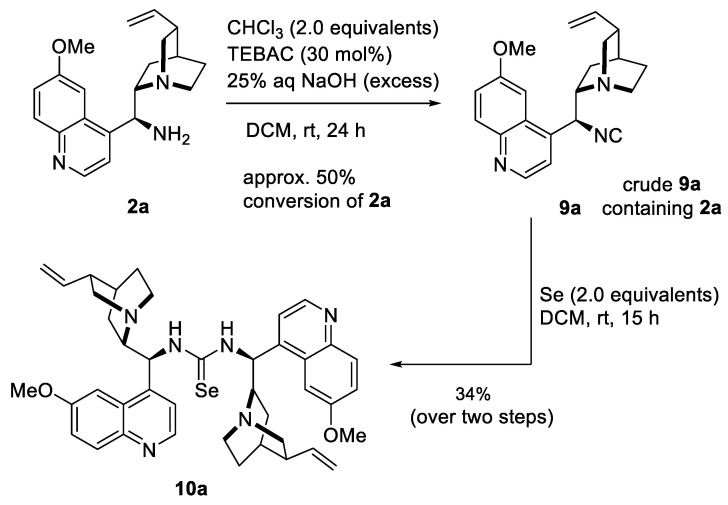
The synthetic route for dimeric alkaloid selenourea **10a**.

Further transformations of the quinine-derived amine **2a** applying the Hofmann isonitrile synthesis protocol [37] modified by Mąkosza [38] followed for the preparation of novel isonitrile **9a**. The reaction of **2a** with chloroform in a biphasic system, 25% aqueous sodium hydroxide solution, and DCM and TEBAC (triethylbenzylammonium chloride) as a phase-transfer catalyst, afforded compound **9a** with a conversion of approximately 50% (Scheme 4). Attempts to isolate detected products were unsuccessful because isocyanide **9a** hydrolyzed during column chromatography with the formation of formamide **8a**.

Accordingly, we turned our attention to converting the resulting crude product into dimeric alkaloid selenourea **10a**. The addition of selenium to the crude mixture of isocyanide **9a** and unreacted amine **2a** in DCM led to a moderate yield of product **10a** (Scheme 4) (spectral data available in Supplementary Materials, Figures S17 and S18).

**Scheme 5:**
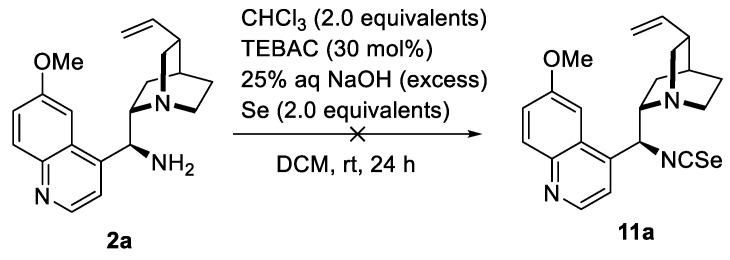
Unsuccessful attempts to prepare isoselenocyanate **11a**.

In contrast, the one-pot transformation of the amine **2a** into isoselenocyanate **11a** under phase-transfer catalysis did not lead to the formation of the desired product **11a** (Scheme 5). In this case, a mixture of compounds was formed.

### 2.2. Catalytic Activity of Tested Compounds

With the aforementioned set of chiral selenoureas in hand, we examined their catalytic abilities as bifunctional organocatalysts in the asymmetric Michael addition. We chose the Michael reaction of nitromethane to *trans*-chalcone developed by Soós et al. [39] as the model reaction.

The screening experiments are summarized in Table 1. The results showed that the selenourea catalysts **7a**–**g** afforded Michael adducts with good to excellent enantioselectivity ranging from 70% to 95% (Table 1, entries 1–7). The influence of substituents in the aromatic ring was also noted for the quinine-based selenourea. As expected, compounds bearing the electron-withdrawing substituent in the phenyl ring delivered a superior effect on the catalyzed reactions in comparison to their counterparts possessing electron-neutral and electron-donating groups. It is known that pKa values of catalysts correlate well with their hydrogen-bond donating abilities [40]. Electron-withdrawing substituents increase the acidity of the double hydrogen bond donor and subsequently, improve the electrophile activation ability [41,42]. There, the application of unsubstituted (entry 1), 4-methoxy- (entry 2), and 4-fluoro-(entry 3) derivatives gave 81, 70, and 95% ee, respectively. Better stereoselectivity was achieved for (8*S*,9*S*)-selenourea derivatives **7c**–**e** (quinine series) as compared to (8*R*,9*R*) quinidine series (Table 1, entries 3–5 vs. entries 6 and 7). These results demonstrate that the configurations of the alkaloid determine the stereochemical result of the addition. The absence of the methoxyl group at the C6′ position in cinchonidine-derived selenourea **7d** did not considerably affect the outcome of the catalyzed reaction compared to the selenourea catalyst **7c**, which possesses this group (91% ee, entry 4 vs. 95% ee, entry 3). The yields of the reaction were poor to moderate (up to 46%), slightly better for selenourea derivatives of the quinine series. Higher yields were observed for dihydro alkaloid-based selenoureas **7e** and **7g** (36–46% ee, entries 5 and 7 vs. 22–26% ee, entries 3,4, and 6).

To directly compare the catalytic utility of sulfur and selenium catalyst counterparts, the sulfur analog **12a** (Figure 1) of the most potent selenourea catalyst **7c** was synthesized (copies of NMR an IR spectra are gathered in Supplementary Materials). When thiourea **12a** was tested a similar result was observed, with slightly lower enantioselectivity (Table 1, entry 8).

All used selenoureas **7a**–**g** decomposed under the reaction conditions to corresponding carbodiimides (noticeable precipitation of black selenium).

**Figure 2:**
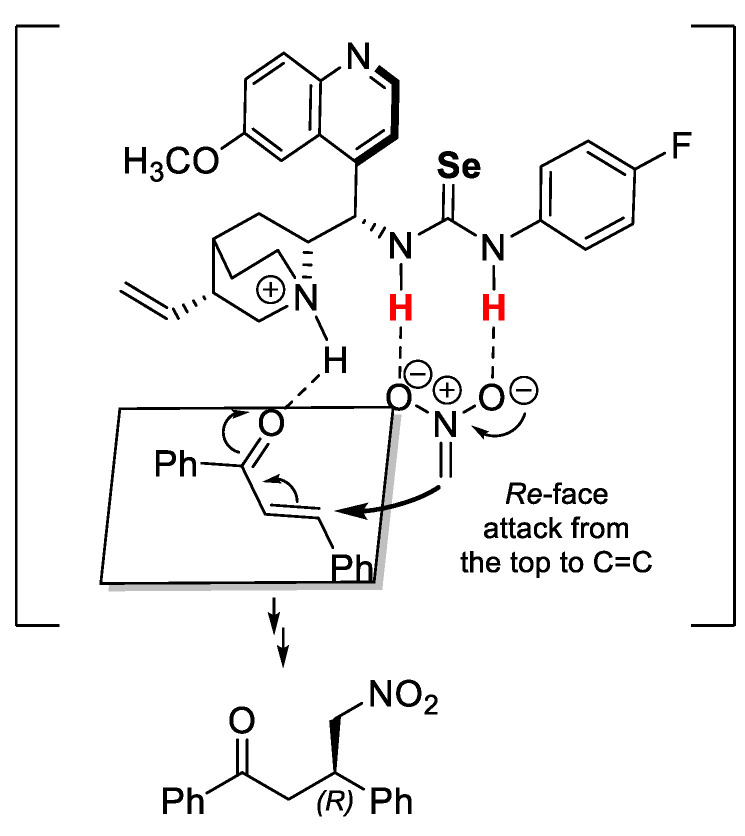
A proposed transition state.

Based on the experimental results and the previous mechanism studies reported by Pedrosa and coworkers [44], a plausible transition state model for the Michael reactions of chalcone **13** and nitromethane catalyzed by **7c** was proposed (Figure 2). In the transition state, the selenourea moiety of the catalyst activates nitromethane by hydrogen bonding while the bridgehead nitrogen atom of the quinine unit activates *trans*-chalcone. While in the transition state, a *Re*-face attack is favored, giving the *R*-configured product.

Next, *Cinchona*-derived selenoureas were applied as organocatalysts for promoting the sulfa-Michael reactions of less reactive thioacetic acid to *trans*-chalcones [45]. All selenourea catalysts afforded the product **15** with high yields ranging from 90% to 99% with up to 21% ee under mild reaction conditions (Table 2). Compounds **7c**–**g**, possessing the 4-fluorophenyl group, were found to be the most potent among probed catalysts. Selenourea **7c** delivered better stereocontrol in the reaction compared to the sulfur counterpart **12a** (Table 2, 21% ee vs. 17% ee, entries 3 and 8). Selenourea catalysts of (8*S*,9*S*) the quinine series **7c**–**e** offered product **15** under better stereocontrol (Table 2, 17–21% ee, entries 3–5 vs. 12–14% ee, entries 6,7). No noticeable catalyst decomposition was observed under the reaction conditions.

The moderate results of conversion (Michael addition) and enantioselectivity (sulfa-Michael addition) indicate that the reaction conditions could be possibly further optimized to improve the catalytic activity of synthesized organocatalysts and to avoid their decomposition (e.g., by altering reaction time and temperature or the used solvent).

## 3. Conclusions

We have developed a synthetic route to the novel achievement of *Cinchona*-based selenoureas and the strategy for producing dimeric alkaloid selenoureas. To our knowledge, this study represents the first example of a promising application of chiral *Cinchona* alkaloid-derived selenoureas as bifunctional organocatalysts in asymmetric Michael addition reactions. Our preliminary attempts resulted in low to excellent enantioselectivities and yields under mild reaction conditions. Further investigations of the catalytic performance in other asymmetric reactions with this type of bifunctional selenoureas are currently underway in our laboratory.

## 4. Materials and Methods

### 4.1. General Information

Solvents were distilled, and other reagents were used as received. Reactions were monitored by thin-layer chromatography (TLC) on silica gel 60 F-254 precoated plates (Merck, Darmstadt, Germany), and spots were visualized with a UV lamp. Products were purified by standard column chromatography on silica gel 60 (230–400 mesh) (Merck). Optical rotations at 578 nm were measured using an Optical Activity Ltd. (Huntington, UK) Model AA-5 automatic polarimeter. Melting points were determined using a Boëtius hot-stage apparatus (PHMK VEB Analytic, Dresden, Germany). ^1^H and ^13^C NMR (600 MHz and 151 MHz, respectively) spectra were recorded in CDCl_3_ on Bruker Avance DRX 300 and NMR Bruker Avance II 600 MHz (Bruker, Billerica, MA, USA). IR spectra were measured using the Vertex 70V vacuum FT-IR spectrometer (Bruker Optics, Ettlingen, Germany). High-resolution mass spectra (HRMS) were recordered using electrospray ionization mode on the Waters LCT Premier XE TOF spectrometer (Waters Corporation, Milford, MA, USA). The enantiomeric ratios of the samples were determined by chiral high-performance liquid chromatography (HPLC) measurements (Thermo Fisher Scientific, Waltham, MA, USA) using Chiracel AD-H or Chiralpak AS-H chiral columns. The configuration of the products was assigned by comparison to literature data.

### 4.2. Preparation of Starting Compounds

The starting *Cinchona* alkaloids (QN, CD, QD, DHQN, DHQD) were commercially available (Buchler GmbH). *Cinchona* alkaloid mesylates were prepared by a standard procedure [26]. 9-Amino-(9-deoxy)-*epi*alkaloids **2a**–**e** were synthesized according to a general procedure described in the literature [33,34]. Aryl isoselenocyanates **6a**–**c** were prepared according to the known procedure [35,46,47].

### 4.3. General Procedure for Selenourea Synthesis

9-Amino-(9-deoxy)-*epi*alkaloid **2** (1.00 mmol, 1.00 equiv.) was dissolved in CH_2_Cl_2_ (5.0 mL), and a solution of appropriate aryl isoselenocyanate **6** (1.00 mmol, 1.00 equiv.) in CH_2_Cl_2_ (1.0 mL) was added. The mixture was stirred for 15 h at rt under argon, in darkness. The solvent was evaporated in vacuo and the residue was purified by chromatography on silica gel (CH_2_Cl_2_/MeOH, 10:1) to afford the desired product **7**. The structure of selenoureas **7** were confirmed by spectroscopic methods (IR, ^1^H, and ^13^C NMR) and high- resolution mass spectrometry (data available in Supplementary Materials).

#### 4.3.1. *N*-[(8*S*,9*S*)-6′-Methoxycinchonan-9-yl]-*N′*-Phenylselenourea *e*QN-**7a**

Yield 85%, pale yellow solid, mp 106–108 °C, R*_f_* = 0.66 (CH_2_Cl_2_/MeOH 10:1).$\;{\left[\alpha \right]}_{D}^{23}\;$= −164.5 (c 0.22, CH_2_Cl_2_). ^1^H NMR (600 MHz, CDCl_3_): δ 9.64 (br s, 1H), 8.44 (s, 1H), 7.97 (d, *J* = 9.1 Hz, 1H), 7.88 (br s, 1H), 7.40–7.34 (m, 3H), 7.32–7.27 (m, 1H), 7.25 (d, *J* = 7.8 Hz, 2H), 7.19 (br s, 1H), 6.15 (br s, 1H), 5.63 (dt, *J* = 17.2, 8.6 Hz, 1H), 4.98−4.90 (m, 2H), 3.96 (s, 3H), 3.52–3.38 (m, 1H), 3.29 (br s, 1H), 3.11 (t, *J* = 12.1 Hz, 1H), 2.75 (d, *J* = 13.9 Hz, 1H), 2.71–2.62 (m, 1H), 2.36–2.20 (m, 1H), 1.75–1.65 (m, 2H), 1.64–1.59 (m, 1H), 1.32 (t, *J* = 11.7 Hz, 1H), 0.97 (dd, *J* = 14.3, 6.4 Hz, 1H) ppm. ^13^C NMR (150 MHz, CDCl_3_): δ 179.2, 157.8, 147.5 (2C overlapped), 144.8, 140.5, 137.5, 131.6, 129.6 (2C overlapped), 128.1, 127.2 (2C overlapped), 125.6, 122.0, 119.9, 115.0, 102.7, 61.0, 58.5, 55.8, 55.1, 41.7, 39.1, 27.4, 27.2, 25.5 ppm. IR: 3157, 2930, 2862, 1620, 1589, 1507, 1473, 1431, 1314, 1297, 1259, 1225, 1028, 915, 850, 835, 823, 758, 693 cm^−1^. HRMS (ESI): m/z calculated for C_27_H_31_N_4_O^80^Se [M+H]^+^: 507.1663, found: 507.1657.

#### 4.3.2. *N*-[(8*S*,9*S*)-6′-Methoxycinchonan-9-yl]-*N′*-(4-Methoxyphenyl)Selenourea *e*QN-**7b**

Yield 82%, pale yellow solid, mp 86–88 °C, R*_f_* = 0.60 (CH_2_Cl_2_/MeOH 10:1).$\;{\left[\alpha \right]}_{D}^{23}\;$= −219.2 (c 0.30, CH_2_Cl_2_). ^1^H NMR (600 MHz, CDCl_3_): δ 8.72 (br s, 1H), 8.59 (s, 1H), 8.00 (d, *J* = 9.0 Hz, 1H), 7.80 (br s, 1H), 7.37 (d, *J* = 9.0 Hz, 1H), 7.25 (br s, 1H), 7.17 (d, *J* = 8.3 Hz, 2H), 6.91 (d, *J* = 8.2 Hz, 2H), 6.02 (br s, 1H), 5.63 (dt, *J* = 17.5, 8.8 Hz, 1H), 5.00–4.91 (m, 2H), 3.96 (s, 3H), 3.83 (s, 3H), 3.42 (br s, 2H), 3.18 (t, *J* = 12.2 Hz, 1H), 2.74 (br s, 2H), 2.31 (s, 1H), 1.78–1.60 (m, 3H), 1.36 (s, 1H), 1.03 (s, 1H) ppm. ^13^C NMR (150 MHz, CDCl_3_): δ 179.1, 158.7, 157.8, 147.5, 145.3, 144.7, 140.9, 131.5, 130.3, 128.2, 127.8 (2C overlapped), 122.1, 119.7, 114.9, 114.8 (2C overlapped), 102.8, 61.1, 58.4, 55.9, 55.6, 55.2, 41.7, 39.3, 27.7, 27.3, 25.6 ppm. IR: 3237, 2931, 2865, 1621, 1507, 1240, 1227, 1166, 1029, 917, 826, 716, 643 cm^−1^. HRMS (ESI): m/z calculated for C_28_H_33_N_4_O_2_^80^Se [M+H]^+^: 537.1771, found: 537.1769.

#### 4.3.3. *N*-(4-Fluorophenyl)-*N′*-[(8*S*,9*S*)-6′-Methoxycinchonan-9-yl]Selenourea *e*QN-**7c**

Yield 86%, pale yellow solid, mp 104–105 °C, R*_f_* = 0.55 (CH_2_Cl_2_/MeOH 10:1).$\;{\left[\alpha \right]}_{D}^{23}\;$= −142.5 (c 0.24, CH_2_Cl_2_). ^1^H NMR (600 MHz, CDCl_3_): δ 9.10 (br s, 1H), 8.57 (s, 1H), 8.00 (d, *J* = 9.2 Hz, 1H), 7.80 (br s, 1H), 7.38 (dd, *J* = 9.2, 2.6 Hz, 1H), 7.28–7.17 (m, 3H), 7.07 (t, *J* = 8.3 Hz, 2H), 6.07 (br s, 1H), 5.64 (dq, *J* = 17.5, 9.5, 8.4 Hz, 1H), 5.02–4.93 (m, 2H), 3.97 (s, 3H), 3.43 (br s, 1H), 3.31 (br s, 1H), 3.16 (t, *J* = 12.1 Hz, 1H), 2.73 (br s, 2H), 2.32 (br s, 1H), 1.72 (br s, 2H), 1.66 (br s, 1H), 1.37 (t, *J* = 12.3 Hz, 1H), 1.00 (br s, 1H) ppm. ^13^C NMR (150 MHz, CDCl_3_) δ 179.7, 161.4 (d, *J*_C–F_ = 247.7 Hz), 158.0, 147.5 (2C overlapped), 144.9, 140.6, 133.7, 131.7, 128.2, 128.1 (2C overlapped), 122.12, 119.7, 116.6 (d, *J*_C–F_ = 22.5 Hz, 2C overlapped), 115.2, 102.8, 61.1, 55.9, 55.2, 50.8, 41.8, 39.2, 27.6, 27.3, 25.6 ppm. IR: 3169, 2931, 2864, 1620, 1504, 1473, 1320, 1217, 1153, 1029, 916, 830, 643 cm^−1^. HRMS (ESI): m/z calculated for C_27_H_30_N_4_O^80^SeF [M+H]^+^: 525.1570, found: 525.1578.

#### 4.3.4. *N*-[(8*S*,9*S*)-Cinchonan-9-yl]-*N′*-(4-Fluorophenyl)Selenourea *e*CD-**7d**

Yield 88%, pale yellow solid, mp. 66–68 °C, R*_f_* = 0.62 (CH_2_Cl_2_/MeOH 10:1). ${\left[\alpha \right]}_{D}^{23}\;$= −173.8 (c 0.32, CH_2_Cl_2_). ^1^H NMR (600 MHz, CDCl_3_): δ 8.93 (s, 1H), 8.78 (s, 1H), 8.47 (s, 1H), 8.13 (d, *J* = 8.4 Hz, 1H), 7.73 (ddd, *J* = 8.4, 6.8, 1.2 Hz, 1H), 7.64 (dd, *J* = 9.7, 5.5 Hz, 1H), 7.35 (s, 1H), 7.30–7.21 (m, 2H), 7.09 (t, *J* = 8.3 Hz, 2H), 6.06 (s, 1H), 5.61 (dq, *J* = 17.8, 8.9, 7.3 Hz, 1H), 5.07–4.82 (m, 2H), 3.42 (m, 2H), 3.26–3.11 (m, 1H), 2.77 (s, 2H), 2.34 (s, 1H), 1.74 (m, 3H), 1.35 (t, *J* = 11.9 Hz, 1H), 1.07 (s, 1H) ppm. ^13^C NMR (150 MHz, CDCl_3_): δ 180.0, 161.3 (d, *J*_C–F_ = 247.5 Hz), 150.0, 148.5, 146.2, 140.2, 133.7, 130.4, 129.4, 127.9 (2C overlapped), 127.0, 126.8, 124.2, 119.7, 116.4 (d, *J*_C–F_ = 22.9 Hz, 2C overlapped), 115.2, 61.2, 58.3, 54.9, 41.5, 38.9, 27.2, 27.1, 25.1 ppm. IR: 3168, 2938, 2864, 1589, 1504, 1320, 1289, 1214, 914, 831, 797, 752, 639 cm^−1^. HRMS (ESI): m/z calculated for C_26_H_28_N_4_^80^SeF [M+H]^+^: 495.1463, found: 495.1463.

#### 4.3.5. *N*-[4-Fluorophenyl]-*N′*-[(8*S*,9*S*)-10,11-Dihydro-6′-Methoxycinchonan-9-yl]Selenourea *e*DHQN-**7e**

Yield 85%, pale yellow solid, mp 62−64 °C, R*_f_* = 0.54 (CH_2_Cl_2_/MeOH 10:1). ${\left[\alpha \right]}_{D}^{23}\;$= −165.7 (c 0.14, CH_2_Cl_2_). ^1^H NMR (600 MHz, CDCl_3_): δ 8.88 (br s, 1H), 8.71 (s, 1H), 8.03 (d, *J* = 9.2 Hz, 1H), 7.89 (br s, 1H), 7.40 (dd, *J* = 9.1, 2.7 Hz, 2H), 7.30 (d, *J* = 7.0 Hz, 2H), 7.05 (t, *J* = 8.3 Hz, 2H), 6.32 (s, 1H), 3.99 (s, 3H), 3.64 (s, 1H), 3.32 (s, 1H), 2.89 (s, 1H), 2.60 (s, 1H), 2.20 (d, *J* = 10.4 Hz, 1H), 1.77 (br m, 4H), 1.45 (s, 1H), 1.24 (q, *J* = 7.9 Hz, 2H), 1.13 (s, 1H), 0.81 (t, *J* = 7.3 Hz, 3H) ppm. (150 MHz, CDCl_3_): δ 180.0, 161.2 (d, *J*_C–F_ = 247.6 Hz), 158.0, 147.5, 144.8, 144.5, 133.9, 131.6, 127.9 (3C overlapped), 122.1, 119.9, 116.3 (d, *J*_C–F_ = 23.2 Hz, 2C overlapped), 102.7, 60.9, 56.6, 55.9, 50.7, 41.9, 36.6, 27.6, 27.0, 25.1, 24.9, 11.8 ppm. IR: 3167, 2929, 2864, 1621, 1505, 1473, 1315, 1218, 1029, 917, 829, 715, 644 cm^−1^. HRMS (ESI): m/z calculated for C_27_H_32_N_4_O^80^SeF [M+H]^+^: 527.1727, found: 527.1732.

#### 4.3.6. *N*-(4-Fluorophenyl)-*N′*-[(8*R*,9*R*)-6′-Methoxycinchonan-9-yl]Selenourea *e*QD-**7f**

Yield 81%, pale yellow solid, mp 71–73 °C, R*_f_* = 0.43 (CH_2_Cl_2_/MeOH 10:1). ${\left[\alpha \right]}_{D}^{23}\;$= +331.1 (c 0.18, CH_2_Cl_2_). ^1^H NMR (600 MHz, CDCl_3_): δ 9.04 (br s, 1H), 8.63 (s, 1H), 8.00 (d, *J* = 9.5 Hz, 1H), 7.77 (br s, 1H), 7.45–7.19 (m, 4H), 7.07 (t, *J* = 8.3 Hz, 2H), 6.17 (s, 1H), 5.90 (dq, *J* = 16.3, 5.9 Hz, 1H), 5.23 (m, 2H), 3.97 (s, 3H), 3.31 (s, 2H), 3.18–2.75 (m, 4H), 2.49–2.24 (m, 1H), 1.58 (m, 2H), 1.36 (s, 1H), 1.03 (s, 1H) ppm. ^13^C NMR (150 MHz, CDCl_3_): δ 180.1, 161.3 (d, *J*_C–F_ = 247.0 Hz), 158.1, 147.5 (2C), 144.9, 139.5, 133.8, 131.7, 128.1, 127.8 (2C overlapped), 122.6, 119.6, 116.4 (d, *J*_C–F_ = 23.3 Hz, 2C overlapped), 115.7, 102.4, 61.3, 55.9, 50.9, 48.8, 47.4, 38.7, 27.2, 26.0, 25.1 ppm. IR: 3169, 2934, 2868, 1620, 1589, 1505, 1473, 1323, 1310, 1319, 1028, 917, 849, 827, 795, 715 cm^−1^. HRMS (ESI): m/z calculated for C_27_H_30_N_4_O^80^SeF [M+H]^+^: 525.1570, found: 525.1575.

#### 4.3.7. *N*-[4-Fluorophenyl]-*N′*-[(8*R*,9*R*)-10,11-Dihydro-6′-Methoxycinchonan-9-yl]Selenourea *e*DHQD-7**g**

Yield 78%, pale yellow solid, mp 88–90 °C, R*_f_* = 0.40 (CH_2_Cl_2_/MeOH 10:1). ${\left[\alpha \right]}_{D}^{23}\;$= +142.5 (c 0.24, CH_2_Cl_2_). ^1^H NMR (600 MHz, CDCl_3_): δ 9.03 (br s, 1H), 8.70 (s, 1H), 8.02 (d, *J* = 9.2 Hz, 1H), 7.89 (br s, 1H), 7.40 (d, *J* = 11.1 Hz, 2H), 7.38–7.30 (m, 2H), 7.04 (t, *J* = 8.5.Hz, 2H), 6.40 (s, 1H), 3.97 (s, 3H), 3.71–3.45 (m, 1H), 3.13 (br m, 3H), 3.03–2.86 (m, 1H), 1.88–1.34 (m, 7H), 1.09 (s, 1H), 0.94 (s, 3H) ppm. ^13^C NMR (150 MHz, CDCl_3_): δ 180.2, 161.1 (d, *J*_C–F_ = 245.5 Hz), 158.0, 147.5, 144.8, 144.2, 133.8, 131.7, 128.0, 127.6 (2C overlapped), 122.4, 119.9, 116.2 (d, *J*_C–F_ = 21.7 Hz, 2C overlapped), 102.3, 61.2, 55.7, 49.5, 48.7, 36.8, 35.0, 26.2, 25.9, 25.3, 24.6, 11.9 ppm. IR: 3181, 2931, 2867, 1621, 1505, 1473, 1322, 1219, 1153, 1029, 848, 829, 715 cm^−1^. HRMS (ESI): m/z calculated for C_27_H_32_N_4_O^80^SeF [M+H]^+^: 527.1727, found: 527.1735.

### 4.4. Preparation of N-[(8S,9S)-6′-Methoxycinchonan-9-yl]Formamide **8a**

Amine **2a** (0.323 g, 1.00 mmol) was placed in a Teflon-capped ampoule and dissolved in an excess of methyl formate (4.00 mL). The mixture was heated to 45 °C for 48 h. The solvent was removed in vacuo, yielding compound **8a** as a solidifying oil quantitatively (0.352 g), pale yellow solid, R*_f_* = 0.25 (CH_2_Cl_2_/MeOH 10:1). ${\left[\alpha \right]}_{D}^{23}$= +32.5° (c 0.20, CH_2_Cl_2_). ^1^H NMR (600 MHz, CDCl_3_): δ 8.73 (dd, *J* = 4.6, 1.8 Hz, 1H), 8.20 (s, 1H), 8.02 (dd, *J* = 9.2, 1.9 Hz, 1H), 7.63 (s, 1H), 7.39 (dt, *J* = 9.2, 2.3 Hz, 1H), 7.33 (d, *J* = 4.6 Hz, 1H), 5.75 (dt, *J* = 17.6, 9.0 Hz, 1H), 5.50 (s, 1H), 5.06–4.97 (m, 2H), 3.98 (s, 4H), 3.36–3.18 (m, 3H), 2.85−2.71 (m, 2H), 2.35 (d, *J* = 7.9 Hz, 2H), 1.72 (q, *J* = 3.0 Hz, 1H), 1.65 (t, *J* = 8.1 Hz, 2H), 1.55 (t, *J* = 12.0 Hz, 1H), 0.94 (dd, *J* = 14.5, 6.2 Hz, 1H) ppm. ^13^C NMR (150 MHz, CDCl_3_): δ 161.0, 158.1, 147.5, 144.9, 144.1, 141.1, 131.8, 128.3, 122.0, 119.1, 114.9, 101.9, 59.5, 56.1, 55.8, 49.6, 41.1, 39.4, 27.7, 27.4, 26.5 ppm. IR: 3226, 3073, 2937, 2864, 1938, 1672, 1621, 1591, 1475, 1434, 1383, 1365, 1229, 1028, 916, 852, 828, 714, 678, 636, 616 cm^−1^. HRMS (ESI): m/z calculated for C_21_H_26_N_3_O_2_ [M+H]^+^: 352.2021, found: 352.2025. 

### 4.5. Preparation of N,N′-bis[(8S,9S)-6′-Metoxycinchonan-9-yl]Selenourea **10a**

9*S*-Amino-9-deoxyquinine **2a** (0.324 g, 1.00 mmol, 1.00 equiv.) was dissolved in CH_2_Cl_2_ (6 mL) and 25% aqueous solution of NaOH (3 mL), triethylbenzylammonium chloride (TEBAC) (68.5 mg, 0.30 mmol, 30.0 mol%) and CHCl_3_ (0.239 g, 160 μL, 2.00 mmol, 2.00 equivalents) were added. The mixture was stirred for 24 h at rt and water (10 mL) was added and the mixture was extracted with CH_2_Cl_2_ (3 × 15 mL). The combined organic phases were dried over Na_2_SO_4_ and evaporated. The residue was dissolved in CH_2_Cl_2_ (10 mL) and selenium (0.158 g, 2.00 mmol, 2.00 equivalents) was added and the solution was stirred overnight under argon. The solvent was removed in vacuo and the residue was purified by chromatography in the dark (silica gel, CH_2_Cl_2_/MeOH 10:1 → MeOH) to afford 0,136 g (37%) of dimeric selenourea **10a** as a pale yellow oil, R*_f_* = 0.05 (CH_2_Cl_2_:MeOH 10:1), ${\left[\alpha \right]}_{D}^{23}\;$= −130.5° (c 0.11, CH_2_Cl_2_). ^1^H NMR (600 MHz, CDCl_3_): δ 8.78 (br s, 1H), 8.67 (br s, 1H), 8.12 (br s, 1H), 7.97 (d, *J* = 9.1 Hz, 1H), 7.49 (br s, 1H), 7.40–7.32 (m, 1H), 6.48 (br s, 1H), 5.58 (br s, 1H), 4.90 (br s, 2H), 3.88 (s, 3H), 3.18 (br s, 1H), 2.90 (br s, 2H), 2.41 (s, 1H), 2.31 (s, 1H), 2.17 (s, 1H), 1.76 (s, 1H), 1.51 (br m, 2H), 1.38 (s, 1H), 0.73 (s, 1H) ppm. IR (KBr): 3250, 2931, 1867, 1621, 1590, 1508, 1473, 1449, 1316, 1261, 1227, 1029, 917, 850, 758, 693, 494 cm^−1^. HRMS (ESI): m/z calculated for C_41_H_49_N_6_O_2_^80^Se [M+H]^+^: 737.3087, found: 737.3089. ^13^C NMR spectrum could not be obtained due to fast decomposition of the compound in solutions.

### 4.6. Preparation of N-(4-Fluorophenyl)-N′-[(8S,9S)-6′-Methoxycinchonan-9-yl]Thiourea eQN-**12a**

To the stirred solution of 9*S*-amino-9-deoxyquinine **2a** (0.323 g, 1.00 mmol) in CH_2_Cl_2_ (5 mL) was added dropwise a solution of 4-fluorophenyl isoselenocyanate **6c** (0.153 g, 1.00 mmol) in CH_2_Cl_2_ (1.00 mL) under nitrogen atmosphere. The resulting mixture was stirred for 15 h. The solvent was removed in vacuo and the residue was purified by column chromatography on silica gel (CH_2_Cl_2_/MeOH 10:1) to afford thiourea 0.286 g (60%) of compound **12** as pale yellow solid, mp 108–110 °C, R*_f_* = 0.45 (CH_2_Cl_2_/MeOH 10:1).$\;{\left[\alpha \right]}_{D}^{23}\;$= −160.8 (c 0.24, CH_2_Cl_2_). ^1^H NMR (600 MHz, CDCl_3_): δ 8.88 (br s, 1H), 8.63 (d, *J* = 4.5 Hz, 1H), 8.18 (br s, 1H), 8.00 (d, *J* = 9.2 Hz, 1H), 7.81 (br s, 1H), 7.38 (dd, *J* = 9.2, 2.6 Hz, 1H), 7.34 (d, *J* = 4.5 Hz, 1H), 7.28–7.23 (m, 2H), 7.03 (t, *J* = 8.5 Hz, 2H), 6.10 (br s, 1H), 5.61 (ddd, *J* = 17.3, 10.2, 7.1 Hz, 1H), 5.04–4.94 (m, 2H), 3.96 (s, 3H), 3.56 (br s, 1H), 3.48 (br s, 1H), 3.24 (dd, *J* = 13.8, 10.2 Hz, 1H), 2.82 (td, *J* = 13.2, 11.5, 5.3 Hz, 2H), 2.38 (d, *J* = 8.6 Hz, 1H), 1.88–1.66 (m, 3H), 1.44 (ddt, *J* = 13.5, 10.2, 3.3 Hz, 1H), 1.02 (dd, *J* = 14.4, 6.2 Hz, 1H) ppm. ^13^C NMR (150 MHz, CDCl_3_): δ 181.2, 160.9 (d, *J* = 246.6 Hz), 158.1, 147.6, 144.8, 144.2, 139.6, 134.0, 131.6, 128.2, 127.3, 122.2, 119.8, 116.1 (d, *J* = 22.6 Hz), 115.7, 102.5, 60.8, 55.9, 55.0, 50.6, 41.9, 38.6, 27.1, 26.8, 25.3 ppm. IR: 3265, 2932, 2879, 1621, 1505, 1473, 1331, 1218, 1029, 990, 917, 831, 716, 551, 507, 452 cm^−1^. HRMS (ESI): m/z calculated for C_27_H_30_N_4_OSF [M+H]^+^: 477.2124, found: 477.2120.

### 4.7. General Procedure for the Michael Addition of Nitromethane to Trans-Chalcones

*Trans*-chalcone **13** (0.104 g, 0.50 mmol, 1.00 equivalent and the respective catalyst (10.0 mol%) were loaded to an ampoule. Next, toluene (0.30 mL) was added and the mixture was stirred for 10 min under argon. Then, nitromethane (91.6 mg, 80.5 μL, 1.50 mmol, 3.00 equivalents) was added by syringe and the reaction was further stirred for 96 h at room temperature. The reaction mixture was directly purified by chromatography (silica gel, hexane/EtOAc 3:1) to afford respective Michael adduct **14**. The enantiomeric excess was determined by HPLC (Chiralcel AD-H column, hexane/*i*-PrOH 9:1, flow rate 1.0 mL/min, λ = 254 nm); t_(S)_ = 14.7 min, t_(R)_ = 20.5 min. The spectral and HPLC data (Supplementary Materials, Figures S21–S29) were in agreement with the reported values [43].

### 4.8. General Procedure for the Sulfa-Michael Addition of Thioacetic Acid to Trans-Chalcone

A reaction tube was loaded with the respective catalyst (10.0 mol%), *trans*-chalcone **13** (52.1 mg, 0.25 mmol, 1.00 equivalent), and diethyl ether (1.25 mL) under argon. After 10 min stirring at rt, thioacetic acid (38.1 mg, 35.2 μL 0.50 mmol, 2.00 equivalents) was added by syringe, and the stirring was continued for 4 h at rt. The reaction mixture was directly purified by chromatography (silica gel, hexane/EtOAc 3:1) to afford respective Michael adduct **15**. Enantiomeric excess values were determined by chiral HPLC (Chiralcel AS-H column, hexane/*i*-PrOH 9:1, flow rate 1.0 mL/min, λ = 254 nm); t_(R)_ = 9.7 min, t_(S)_ = 15.0 min. The spectral and HPLC data (Supplementary Materials, Figures S30–S38) were in agreement with the reported values [44].

## Figures and Tables

**Table 1:**
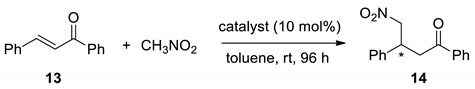
Asymmetric Michael addition of nitromethane to *trans*-chalcone ^a^.

Entry	Catalyst	Yield (%) ^b^	ee (%) ^c^	Config. ^d^
1	*e*QN-7**a**	21	81	*R*
2	*e*QN-7**b**	16	70	*R*
3	*e*QN-7**c**	26	95	*R*
4	*e*CD-7**d**	25	91	*R*
5	*e*DHQN-7**e**	46	95	*R*
6	*e*QD-7**f**	22	87	*S*
7	*e*DHQD-7**g**	36	86	*S*
8	*e*QN-12**a**	24	89	*R*

^a^ The reaction was carried out on a 0.5 mmol scale of **13** and 3.0 equiv. of nitromethane in toluene (0.3 mL), 10 mol% of catalyst in capped ampoules at rt for four days. ^b^ Isolated product yield. ^c^ Determined by chiral-phase HPLC using an AD-H column. ^d^ Determined by comparison with available literature HPLC data [43].

**Table 2:**
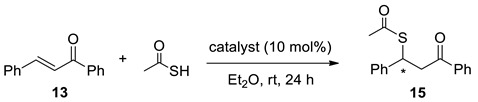
Sulfa-Michael addition of thioacetic acid to *trans*-chalcone ^a^.

Entry	Catalyst	Yield (%) ^b^	ee (%) ^c^	Config. ^d^
1	*e*QN-7**a**	92	13	*R*
2	*e*QN-7**b**	90	3	*R*
3	*e*QN-7**c**	93	21	*R*
4	*e*CD-7**d**	95	17	*R*
5	*e*DHQN-7**e**	99	21	*R*
6	*e*QD-7**f**	94	14	*R*
7	*e*DHQD-7**g**	98	12	*R*
8	*e*QN-12**a**	89	17	*R*

^a^ The reaction was carried out using **13** (0.25 mmol) and thioacetic acid (0.5 mmol) in the presence of 10 mol% catalyst in 1.25 mL of Et_2_O at rt for 4 h. ^b^ Isolated yield. ^c^ Determined by chiral-phase HPLC using an AS-H column. ^d^ Determined by comparison with available literature HPLC data [45].

## Data Availability

The data are available on request from the corresponding author.

## Supplementary Materials

The following are available online at https://www.mdpi.com/1996-1944/14/3/600/s1. Figure S1. 1H and 13C NMR spectra of *N*-[(8*S*,9*S*)-6′-methoxycinchonan-9-yl]-*N′*-phenylselenourea *e*QN-7a, Figure S2. IR spectra of *N*-[(8*S*,9*S*)-6′-methoxycinchonan-9-yl]-*N′*-phenylselenourea *e*QN-7a, Figure S3. 1H and 13C NMR spectra of *N*-[(8*S*,9*S*)-6′-methoxycinchonan-9-yl]-*N*′-[4-methoxyphenyl]selenourea *e*QN-7b, Figure S4. IR spectra of *N*-[(8*S*,9*S*)-6′-methoxycinchonan-9-yl]-*N′*-[4-methoxyphenyl]selenourea *e*QN-7b, Figure S5. 1H and 13C NMR spectra of *N*-[4-fluorophenyl]-*N′*-[(8*S*,9*S*)-6′-methoxycinchonan-9-yl]selenourea *e*QN-7c, Figure S6. IR spectra of *N*-[4-fluorophenyl]-*N′*-[(8*S*,9*S*)-6′-methoxycinchonan-9-yl]selenourea *e*QN-7c, Figure S7. 1H and 13C NMR spectra of *N*-[4-fluorophenyl]-*N′*-[(8*S*,9*S*)-cinchonan-9-yl]selenourea *e*CD-7d, Figure S8. IR spectra of *N*-[4-fluorophenyl]-*N′*-[(8*S*,9*S*)-cinchonan-9-yl]selenourea *e*CD-7d, Figure S9. 1H and 13C NMR spectra of *N*-[4-fluorophenyl]-*N′*-[(8*S*,9*S*)-10,11-dihydro-6′-methoxycinchonan -9-yl]selenourea *e*DHQN-7e, Figure S10. IR spectra of *N*-[4-fluorophenyl]-*N′*-[(8*S*,9*S*)-10,11-dihydro-6′-methoxycinchonan-9-yl]selenourea *e*DHQN-7e, Figure S11. 1H and 13C NMR spectra of *N*-[4-fluorophenyl]-*N′*-[(8*R*,9*R*)-6′-methoxycinchonan -9-yl]selenourea *e*QD-7f, Figure S12. IR spectra of *N*-[4-fluorophenyl]-*N′*-[(8*R*,9*R*)-6′- methoxycinchonan-9-yl]selenourea *e*QD-7f, Figure S13. 1H and 13C NMR spectra of *N*-[4-fluorophenyl]-*N′*-[(8*R*,9*R*)-10,11-dihydro-6′-methoxycinchonan-9-yl]selenourea *e*DHQD-7g, Figure S14. IR spectra of *N*-[4-fluorophenyl]-*N′*-[(8*R*,9*R*)-10,11-dihydro-6′-methoxycinchonan-9-yl]selenourea *e*DHQD-7g, Figure S15. 1H and 13C NMR spectra of *N*-[(8*S*,9*S*)-6′-methoxycinchonan-9-yl]formamide 8a, Figure S16. IR spectra of *N*-[(8*S*,9*S*)-6′-methoxycinchonan-9-yl]formamide 8a, Figure S17. 1H NMR spectra of *N,N′*-bis[(8*S*,9*S*)-6′-metoxycinchonan-9-yl]selenourea 10a, Figure S18. IR spectra of *N,N′*-bis[(8*S*,9*S*)-6′- metoxycinchonan-9-yl]selenourea 10a, Figure S19. 1H and 13C NMR spectra of *N*-[4-fluorophenyl]-*N′*-[(8*S*,9*S*)-6′-methoxycinchonan-9-yl]thiourea *e*QN-12a, Figure S20. IR spectra of *N*-[4-fluorophenyl]-*N′*-[(8*S*,9*S*)-6′-methoxycinchonan-9-yl]thiourea *e*QN-12a, Figure S21. HPLC chromatogram for 14: sample obtained with catalyst *e*QN-7a, Figure S22. HPLC chromatogram for 14: sample obtained with catalyst *e*QN-7b, Figure S23. HPLC chromatogram for 14: sample obtained with catalyst *e*QN-7c, Figure S24. HPLC chromatogram for 14: sample obtained with catalyst *e*CD-7d, Figure S25. HPLC chromatogram for 14: sample obtained with catalyst *e*DHQN-7e, Figure S26. HPLC chromatogram for 14: sample obtained with catalyst *e*QD-7f, Figure S27. HPLC chromatogram for 14: sample obtained with catalyst *e*DHQD-7g, Figure S28. HPLC chromatogram for 14: sample obtained with catalyst *e*QN-12a, Figure S29. HPLC chromatogram for racemic 14 without catalyst 7, Figure S30. HPLC chromatogram for 15: sample obtained with catalyst *e*QN-7a, Figure S31. HPLC chromatogram for 15: sample obtained with catalyst *e*QN-7b, Figure S32. HPLC chromatogram for 15: sample obtained with catalyst *e*QN-7c, Figure S33. HPLC chromatogram for 15: sample obtained with catalyst *e*CD-7d, Figure S34. HPLC chromatogram for 15: sample obtained with catalyst *e*DHQN-7e, Figure S35. HPLC chromatogram for 15: sample obtained with catalyst *e*QD-7f, Figure S36. HPLC chromatogram for 15: sample obtained with catalyst *e*DHQD-7g, Figure S37. HPLC chromatogram for 15: sample obtained with catalyst *e*QN-12a, Figure S38. HPLC chromatogram for racemic 15 without catalyst 7.

## References

[1] Bondi A. (1964). Van der Waals volumes and radii. J. Phys. Chem..

[2] Bharatam P.V., Moudgil R., Kaur D. (2003). Electron delocalization in isocyanates, formamides, and ureas: Importance of orbital interactions. J. Phys. Chem. A.

[3] Bibelayi D., Lundemba A.S., Allen F.H., Galek P.T.A., Pradon J., Reilly A.M., Groomb C.R., Yav Z.G. (2016). Hydrogen bonding at C=Se acceptors in selenoureas,selenoamides and selones. Acta Cryst..

[4] Merino-Montiel P., Maza S., Martos S., López Ó., Maya I., Fernández-Bolaños J.G. (2013). Synthesis and antioxidant activity of *O*-alkyl selenocarbamates, selenoureas and selenohydantoins. Eur. J. Pharm. Sci..

[5] Ruberte A.C., Ramos-Inza S., Aydillo C., Talavera I., Encío I., Plano D., Sanmartín C. (2020). Novel *N*,*N′*-disubstituted acylselenoureas as potential antioxidant and cytotoxic agents. Antioxidants.

[6] Hussain R.A., Badshah A., Tahir M.N., Hassan T.U., Bano A. (2014). Synthesis, chemical characterization, DNA binding, antioxidant, antibacterial, and antifungal activities of ferrocence incorporated selenoureas. J. Biochem. Mol. Toxicol..

[7] Díaz M., de Lucio H., Moreno E., Espuelas S., Aydillo C., Jiménez-Ruiz A., Toro M.A., Gutiérrez K.J., Martínez-Merino V., Cornejo A. (2019). Novel urea, thiourea and selenourea derivatives of diselenides: Synthesis and leishmanicidal activity. Antimicrob. Agents Chemoth..

[8] Zakrzewski J., Krawczyk M. (2009). Synthesis and pesticidal properties of thio and seleno analogs. Phosphorus Sulfur Silicon Relat. Elem..

[9] Sivapriya K., Suguna P., Banerjee A., Saravanan V., Rao D.N., Chandrasekaran S. (2007). Facile one-pot synthesis of thio and selenourea derivatives: A new class of potent urease inhibitors. Bioorg. Med. Chem. Lett..

[10] Hussain R.A., Badshah A., Shah A. (2014). Synthesis and biological applications of selenoureas. Appl. Organomet. Chem..

[11] Alcolea V., Plano D., Karelia D.N., Palop J.A., Amin S., Sanmartín C., Sharma A.K. (2016). Novel seleno- and thio-urea derivatives with potent in vitro activities against several cancer cell lines. Eur. J. Med. Chem..

[12] Koketsu M., Ishihara H. (2003). Synthesis of 1,3-selenazine and 1,3-selenazole and their biological activities. Curr. Org. Chem..

[13] Ninomiya M., Garud D.R., Koketsu M. (2010). Selenium-containing heterocycles using selenoamides, selenoureas, selenazadines, and isoselenocyanates. Heterocycles.

[14] Casula A., Llopis-Lorente A., Garau A., Isaia F., Kubicki M., Lippolis V., Sancenon F., Martinez-Manez R., Owczarzak A., Santi C. (2017). A new class of silica-supported chromo-fluorogenic chemosensors for anion recognition based on a selenourea scaffold. Chem. Commun..

[15] Picci G., Mocci R., Ciancaleoni G., Lippolis V., Zielińska-Błajet M., Caltagirone C. (2020). Bis-selenoureas for anion binding: A 1H NMR and theoretical study. ChemPlusChem..

[16] López Ó., Maza S., Ulgar V., Maya I., Fernández-Bolaños J.G. (2009). Synthesis of sugar-derived isoselenocyanates, selenoureas, and selenazoles. Tetrahedron.

[17] Chennakrishnareddy G., Nagendra G., Hemantha H.P., Das U., Guru Row T.N., Sureshbabu V., Chennakrishnareddy G., Nagendra G., Hemantha H.P., Das U. (2010). Isoselenocyanates derived from Boc/Z-amino acids: Synthesis, isolation, characterization, and application to the efficient synthesis of unsymmetrical selenoureas and selenoureidopeptidomimetics. Tetrahedron.

[18] Bian G., Yang S., Huang H., Zong H., Song L., Fan H., Sun X. (2016). Chirality sensing of tertiary alcohols by a novel strong hydrogen-bonding donor—selenourea. Chem. Sci..

[19] Jörres M., Schiffers I., Atodiresei I., Bolm C. (2012). Asymmetric Michael additions of α–nitrocyclohexanone to aryl nitroalkenes catalyzed by natural amino acid-derived bifunctional thioureas. Org. Lett..

[20] Connon S.J. (2008). Asymmetric catalysis with bifunctional cinchona alkaloid-based urea and thiourea organocatalysts. Chem. Commun..

[21] Lu L.Q., An L.Q., Chen J.R., Xiao W.J. (2012). Dual activation in organocatalysis: Design of tunable and bifunctional organocatalysts and their applications in enantioselective reactions. Synlett.

[22] Serdyuk O.V., Heckel C.M., Tsogoeva S. (2013). Bifunctional primary amine-thioureas in asymmetric organocatalysis. Org. Biomol. Chem..

[23] Parvin T., Yadava R., Choudhury L.H. (2020). Recent applications of thiourea-based organocatalysts in asymmetric multicomponent reactions (AMCRs). Org. Biomol. Chem..

[24] Steppeler F., Iwan D., Wojaczyńska E., Wojaczyński J. (2020). Chiral thioureas–preparation and significance in asymmetric synthesis and medicinal chemistry. Molecules.

[25] Song C.E. (2009). Cinchona Alkaloids in Synthesis and Catalysis: Ligands, Immobilization and Organocatalysis.

[26] Ishihara H., Koketsu M., Fukuta Y., Nada F. (2001). Reaction of lithium aluminum hydride with elemental selenium:  its application as a selenating reagent into organic molecules. J. Am. Chem. Soc..

[27] Zhou Y., Denk M.K. (2003). Synthesis and reactivity of subvalent compounds. Part 13: Reaction of triethyl orthoformate with amines and selenium—a convenient one-step three-component synthesis for selenoureas. Tetrahedron Lett..

[28] Koketsu M., Takakura N., Ishihara H. (2002). Efficient synthesis of selenoureas from the corresponding carbodiimides. Synth Commun..

[29] Takikawa Y., Watanabe H., Sasaki R., Shimada K. (1994). Conversion of amides, *N*,*N*,*N*′,*N*′-tetramethylurea, and esters to the corresponding selenoxo compounds by treatment with bis(trimethylsilyl) selenide and BF_3_·OEt_2_. Bull. Chem. Soc. Jpn..

[30] Fernández-Bolaños J.G., López Ó., Ulgar V., Maya I., Fuentes J. (2004). Synthesis of *O*-unprotected glycosyl selenoureas. A new acces to bicyclic sugar isoureas. Tetrahedron Lett..

[31] Zakrzewski J., Huras B., Kiełczewska A. (2016). Synthesis of isoselenocyanates. Synthesis.

[32] Zielińska-Błajet M., Kucharska M., Skarżewski J. (2006). Simple preparation of enantiomeric Michael adducts of thiophenol to chalcones: Easily available new chiral building blocks. Synthesis.

[33] Kacprzak K., Gierczyk B. (2010). Clickable 9-azido-(9-deoxy)-*Cinchona* alkaloids: Synthesis and conformation. Tetrahedron Asymmetry.

[34] Brunner H.J., Bügler H.J., Nuber B. (1995). Preparation of 9-amino(9-deoxy)cinchona alkaloids. Tetrahedron Asymmetry.

[35] Sharma S., Maurya R.A., Min K.I., Jeong G.Y., Kim D.P. (2013). Odorless isocyanide chemistry: An integrated microfluidic system for a multistep reaction sequence. Angew. Chem. Int. Ed. Engl..

[36] Walborsky H.M., Niznik G.E. (1972). Synthesis of isonitriles. J. Org. Chem..

[37] Hofmann A.W. (1868). Ueber eine neue reihe von homologen der cyanwasserstoffsäure. Liebigs Ann. Chem..

[38] Mąkosza M., Wawrzyniewicz M. (1969). Reactions of organic anions. XXIV. Catalytic method for preparation of dichlorocyclopropane derivatives in aqueous medium. Tetrahedron Lett..

[39] Vakulya B., Varga S., Csámpai A., Soós T. (2005). Highly enantioselective conjugate addition of nitromethane to chalcones using bifunctional Cinchona organocatalysts. Org. Lett..

[40] Li X., Deng H., Zhang B., Li J., Zhang L., Luo S., Cheng J.P. (2010). Physical organic study of structure–activity– enantioselectivity relationships in asymmetric bifunctional thiourea catalysis: Hints for the design of new organocatalysts. Chem. Eur. J..

[41] Jakab G., Tancon C., Zhang Z., Lippert K.M., Schreiner P.R. (2012). (Thio)urea organocatalyst equilibrium acidities in DMSO. Org. Lett..

[42] Zhang Z., Bao Z., Xing H. (2014). *N*,*N*′-Bis[3,5-bis(trifluoromethyl)phenyl]thiourea: A privileged motif for catalyst development. Org. Biomol. Chem..

[43] Corey E.J., Zhang F.Y. (2000). Enantioselective Michael addition of nitromethane to alpha, beta-enones catalyzed by chiral quaternary ammonium salts. A simple synthesis of (*R*)-baclofen. Org. Lett..

[44] Manzano R., Andrés J.M., Álvarez R., Muruzábal M.D., de Lera Á.R., Pedrosa R. (2011). Enantioselective conjugate addition of nitro compounds to α,β-unsaturated ketones: An experimental and computational study. Chem. Eur. J..

[45] Li H., Zu L., Wang J., Wang W. (2006). Organocatalytic enantioselective Michael addition of thioacetic acid to enones. Tetrahedron Lett..

[46] Barton D.H.R., Parekh S.I., Tajbakhsh M., Theodorakis E.A., Chi-Lam T. (1994). A convenient and high yielding procedure for the preparation of isoselenocyanates. Synthesis and reactivity of *O*-alkylselenocarbamates. Tetrahedron.

[47] Perez-Labrada K., Brouard I., . Mendez I., Rivera D.G. (2012). Multicomponent synthesis of Ugi-type ceramide analogues and neoglycolipids from lipidic isocyanides. J. Org. Chem..

