# Supplementary material for: Novel Selenoureas Based on Cinchona Alkaloid Skeleton: Synthesis and Catalytic Investigations
Source: Materials (Basel). 2021 Jan 28;14(3):600. doi: 10.3390/ma14030600 (PMC7866029; doi:10.3390/ma14030600)
Supplement: Supplementary file 1 [file materials-14-00600-s001.pdf]

materials

MDPI

Supplementary Materials

Novel Selenoureas Based on *Cinchona* Alkaloid Skeleton: Synthesis and Catalytic Investigations

Mariola Zielińska-Błajet [1,*] and Joanna Najdek [2]

[1] Faculty of Chemistry, Wrocław University of Science and Technology, Wybrzeże Wyspiańskiego 27, 50-370 Wrocław, Poland; mariola.zielinska-blajet@pwr.edu.pl
[2] Institute of Chemistry and Biochemistry, Freie Universität Berlin, Takustr. 3, 14195 Berlin, Germany; joanna.najdek@hotmail.com
* Correspondence: mariola.zielinska-blajet@pwr.edu.pl; Tel.: + 48-71-3202128

1. Spectral Data

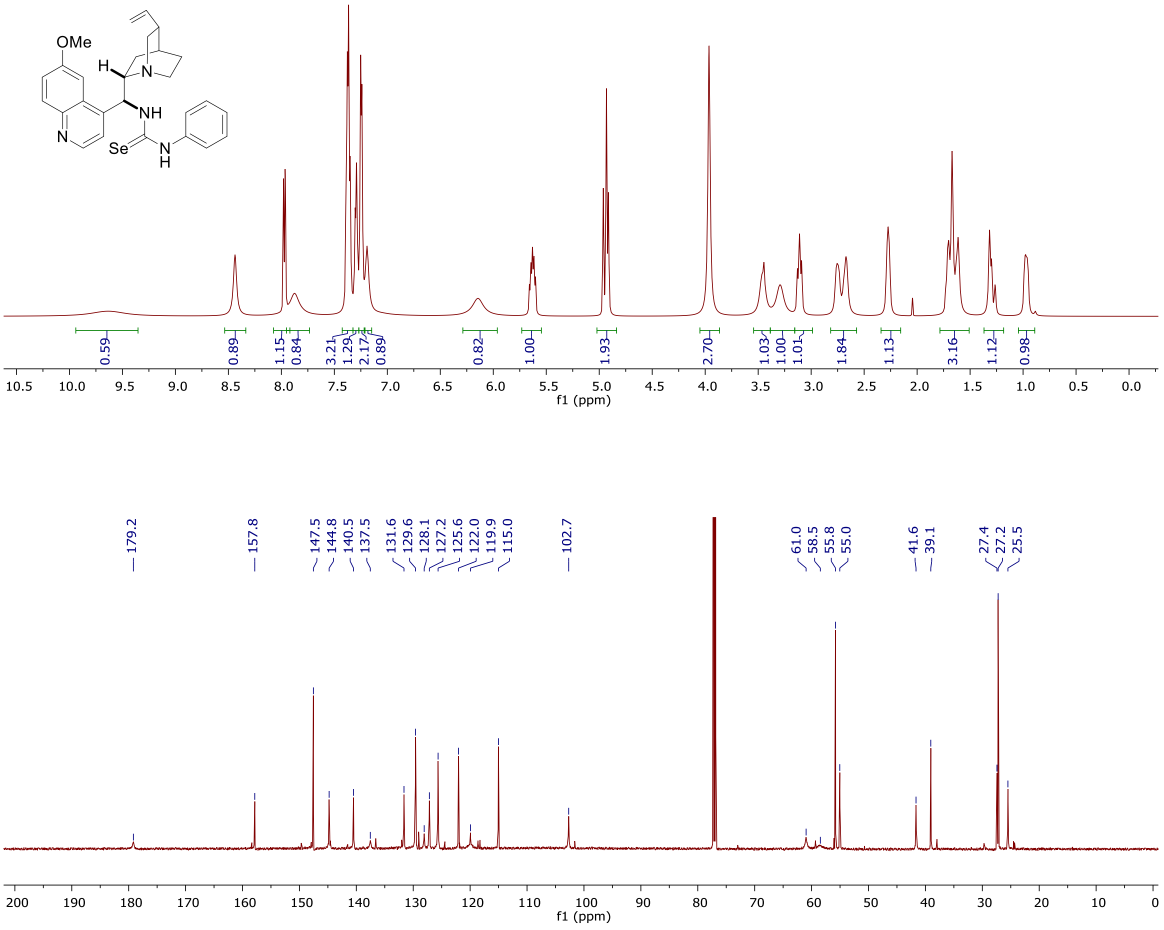

Figure S1. 1H and 13C NMR spectra of *N*-[(8*S*,9*S*)-6′-methoxycinchonan-9-yl]-*N*′-phenylselenourea *e*QN-**7a**.

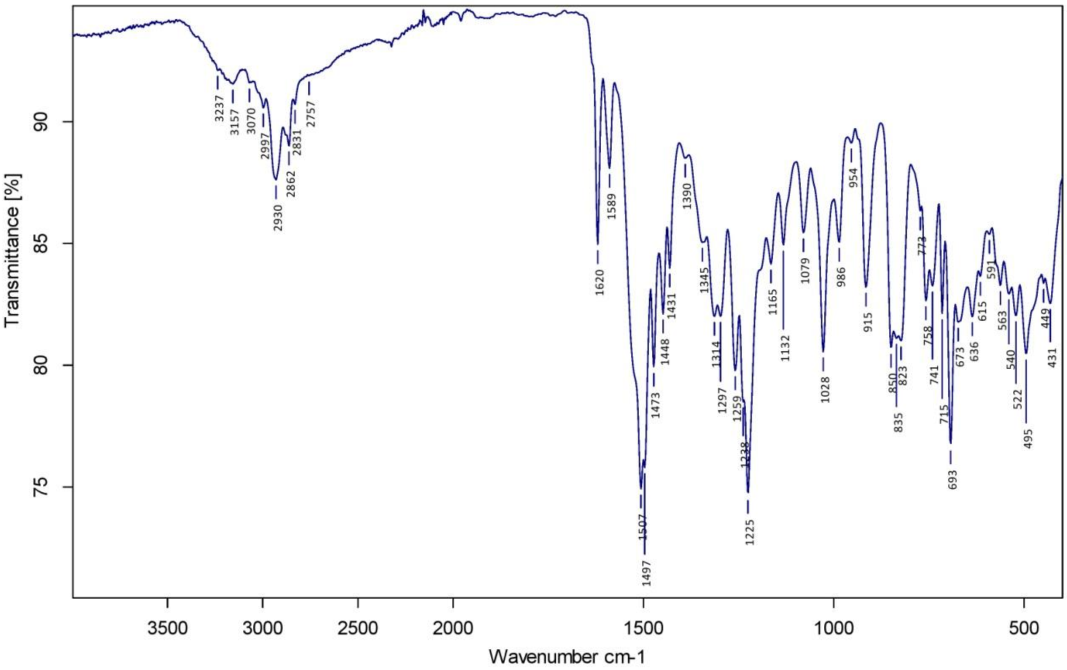

Figure S2. IR spectra of *N*-[(8*S*,9*S*)-6′-methoxycinchonan-9-yl]-*N*′-phenylselenourea *e*QN-**7a**.

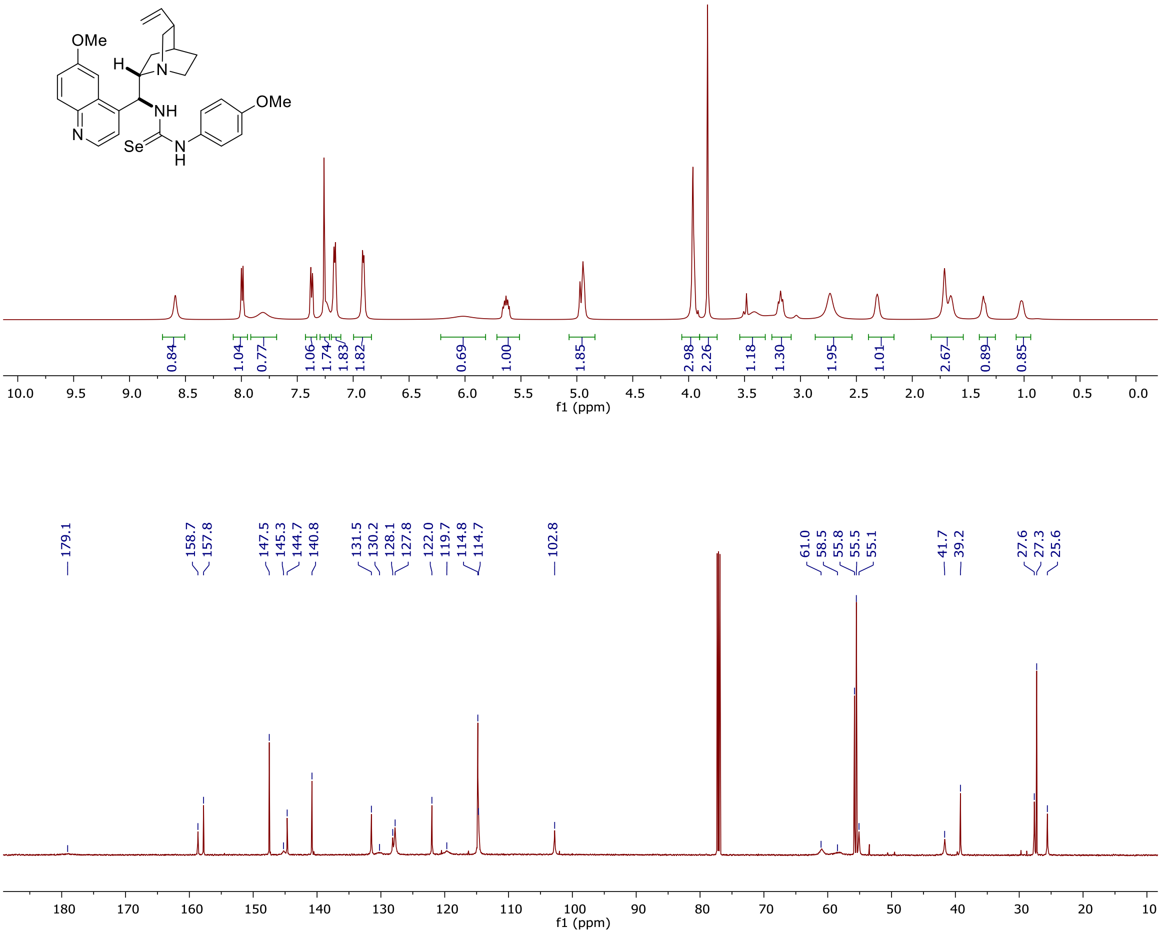

Figure S3. 1H and 13C NMR spectra of *N*-[(8*S*,9*S*)-6′-methoxycinchonan-9-yl]-*N*′-[4- methoxyphenyl]selenourea *e*QN-**7b**.

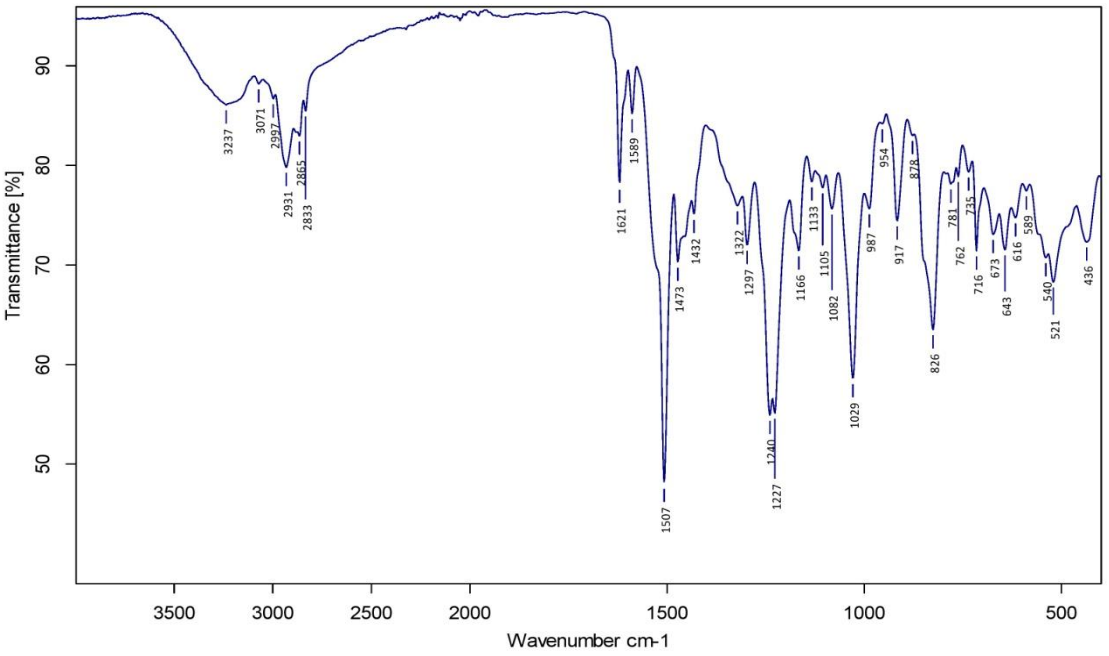

Figure S4. IR spectra of *N*-[(8*S*,9*S*)-6′-methoxycinchonan-9-yl]-*N*′-[4-methoxyphenyl]selenourea *e*QN-**7b**.

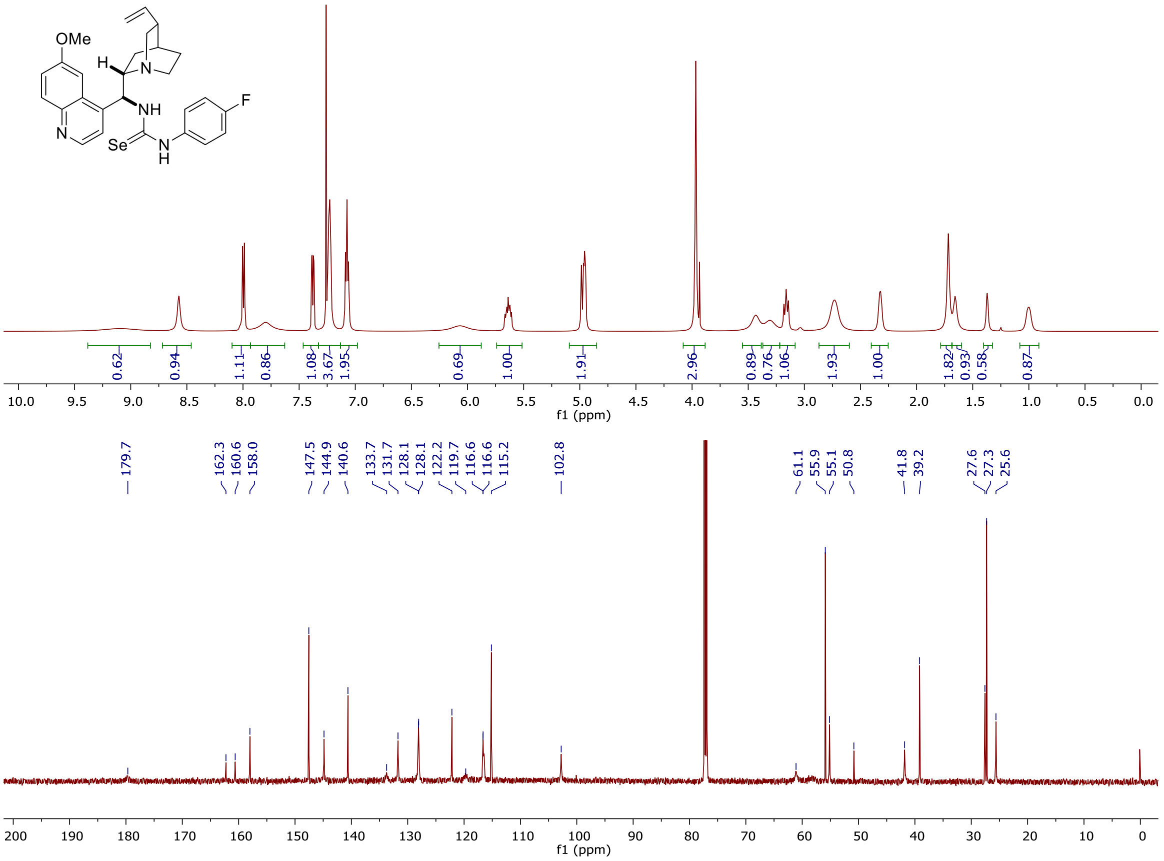

Figure S5. 1H and 13C NMR spectra of *N*-[4-fluorophenyl]-*N*′-[(8*S*,9*S*)-6′-methoxycinchonan-9-yl]selenourea *e*QN-**7c**.

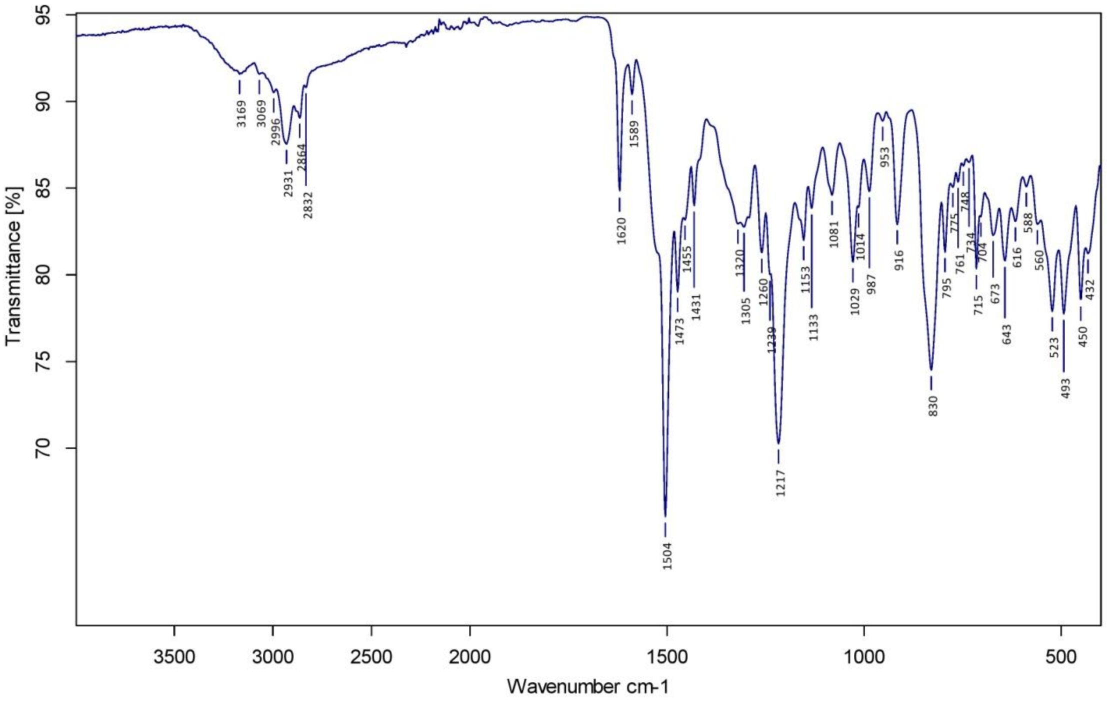

Figure S6. IR spectra of *N*-[4-fluorophenyl]-*N'*-[(8*S*,9*S*)-6'-methoxycinchonan-9-yl]selenourea *e*QN-**7c**.

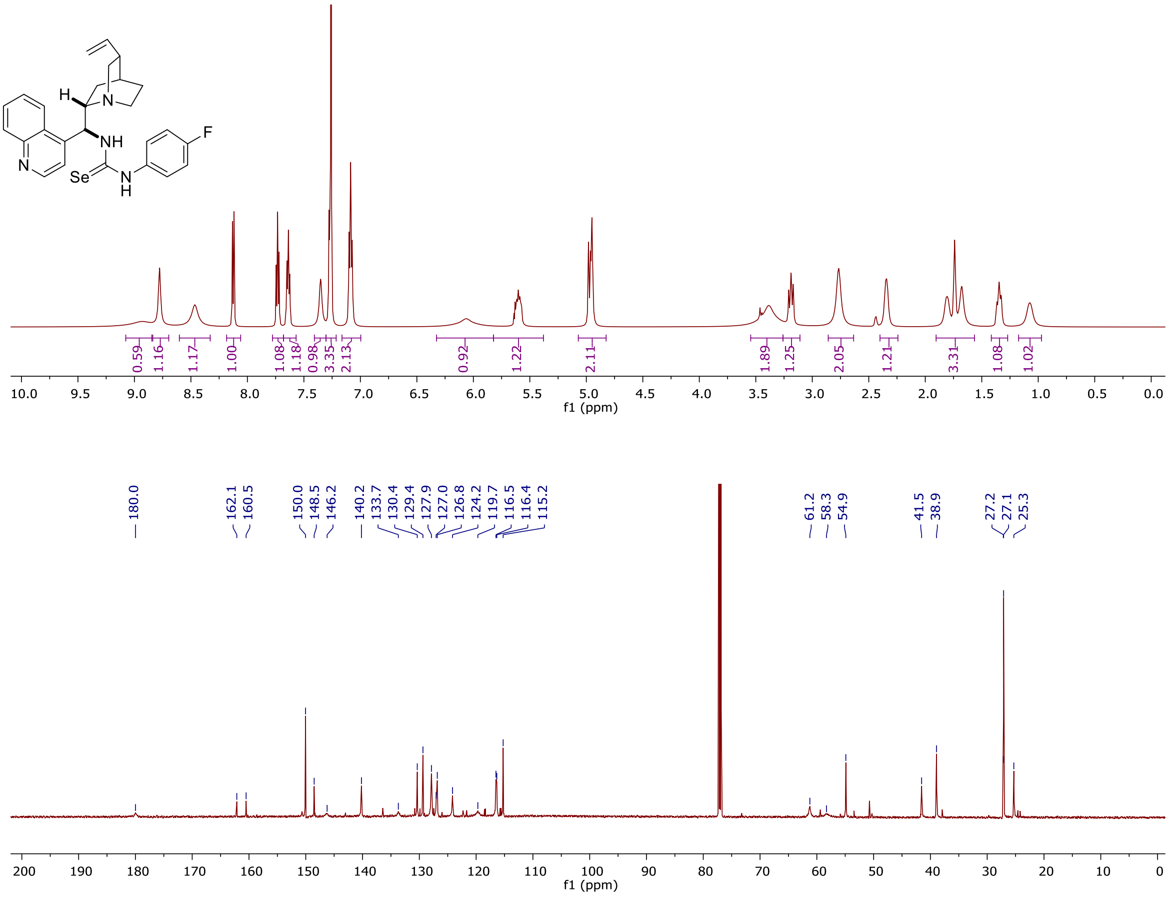

Figure S7. 1H and 13C NMR spectra of *N*-[4-fluorophenyl]-*N'*-[(8*S*,9*S*)-cinchonan-9-yl]selenourea *e*CD-**7d**.

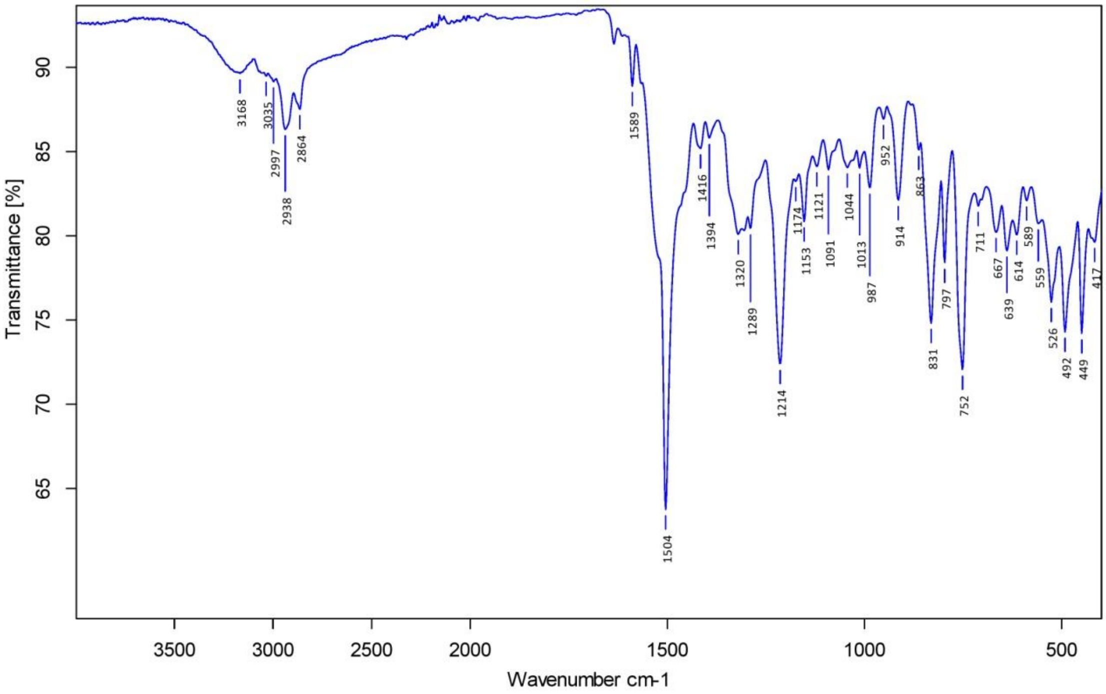

Figure S8. IR spectra of *N*-[4-fluorophenyl]-*N′*-[(8*S*,9*S*)-cinchonan-9-yl]selenourea *e*CD-**7d**.

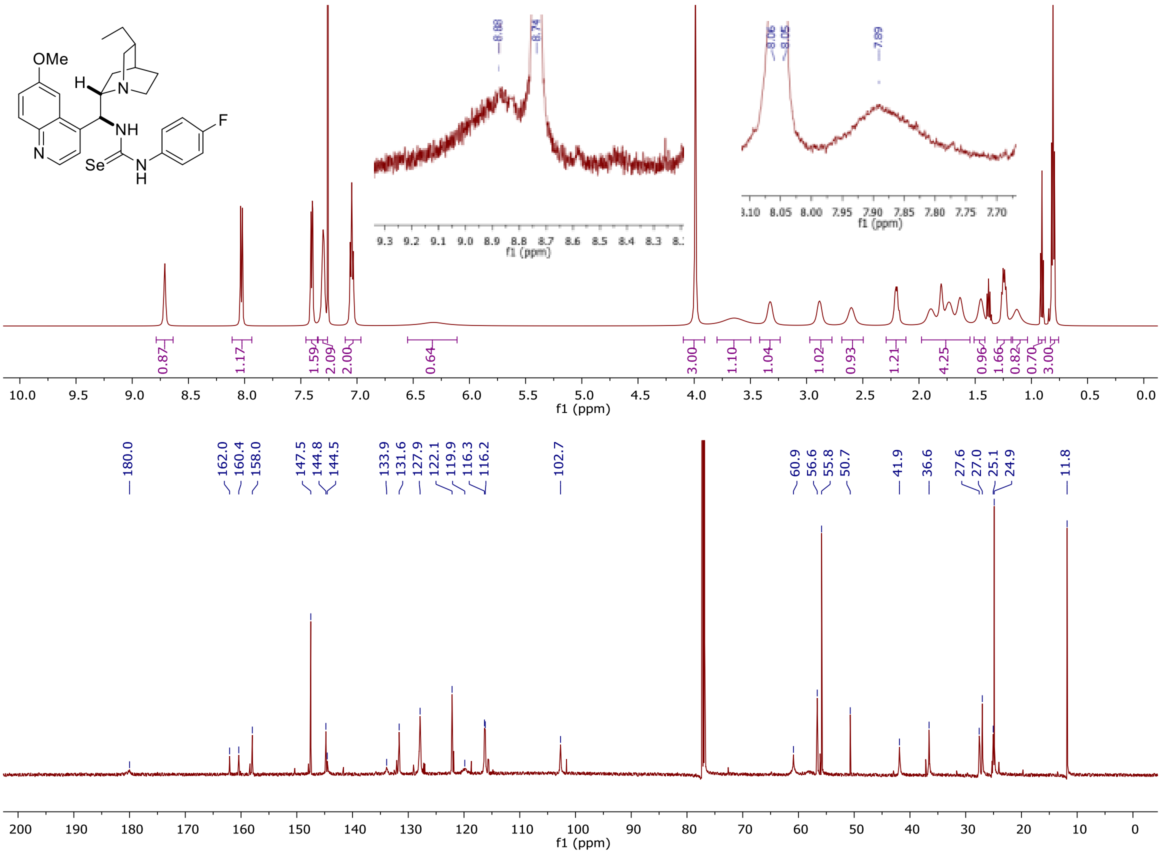

Figure S9. 1H and 13C NMR spectra of *N*-[4-fluorophenyl]-*N′*-[(8*S*,9*S*)-10,11-dihydro-6′-methoxycinchonan -9-yl]selenourea *e*DHQN-**7e**.

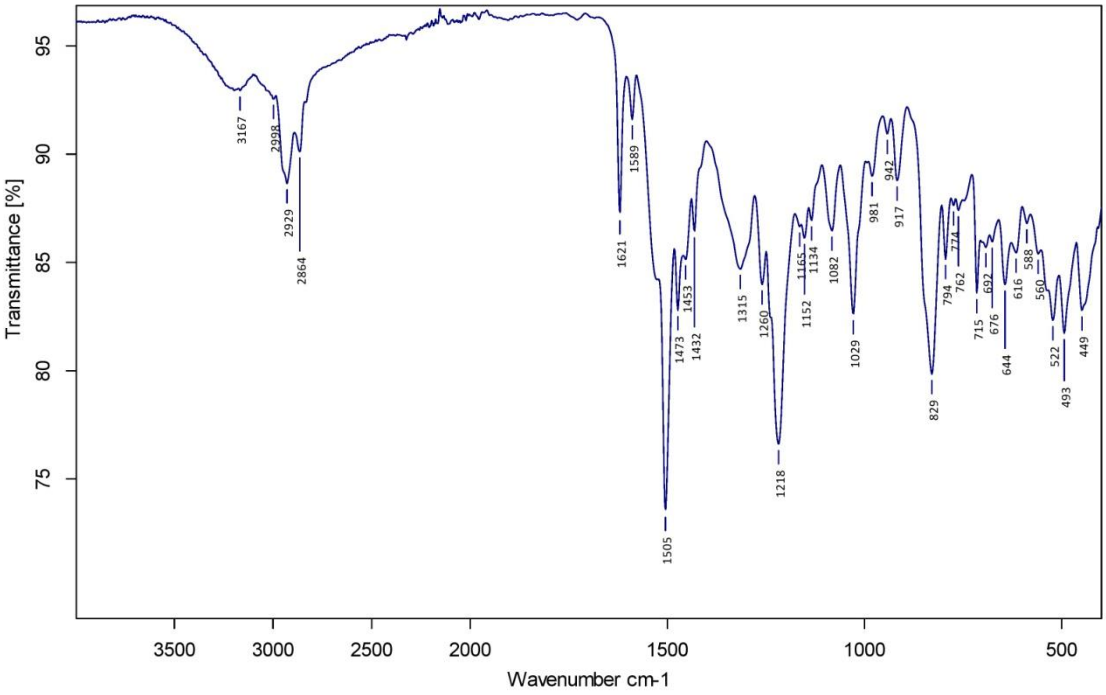

Figure S10. IR spectra of *N*-[4-fluorophenyl]-*N*′-[(8*S*,9*S*)-10,11-dihydro-6′-methoxycinchonan-9-yl]selenourea *e*DHQN-**7e**.

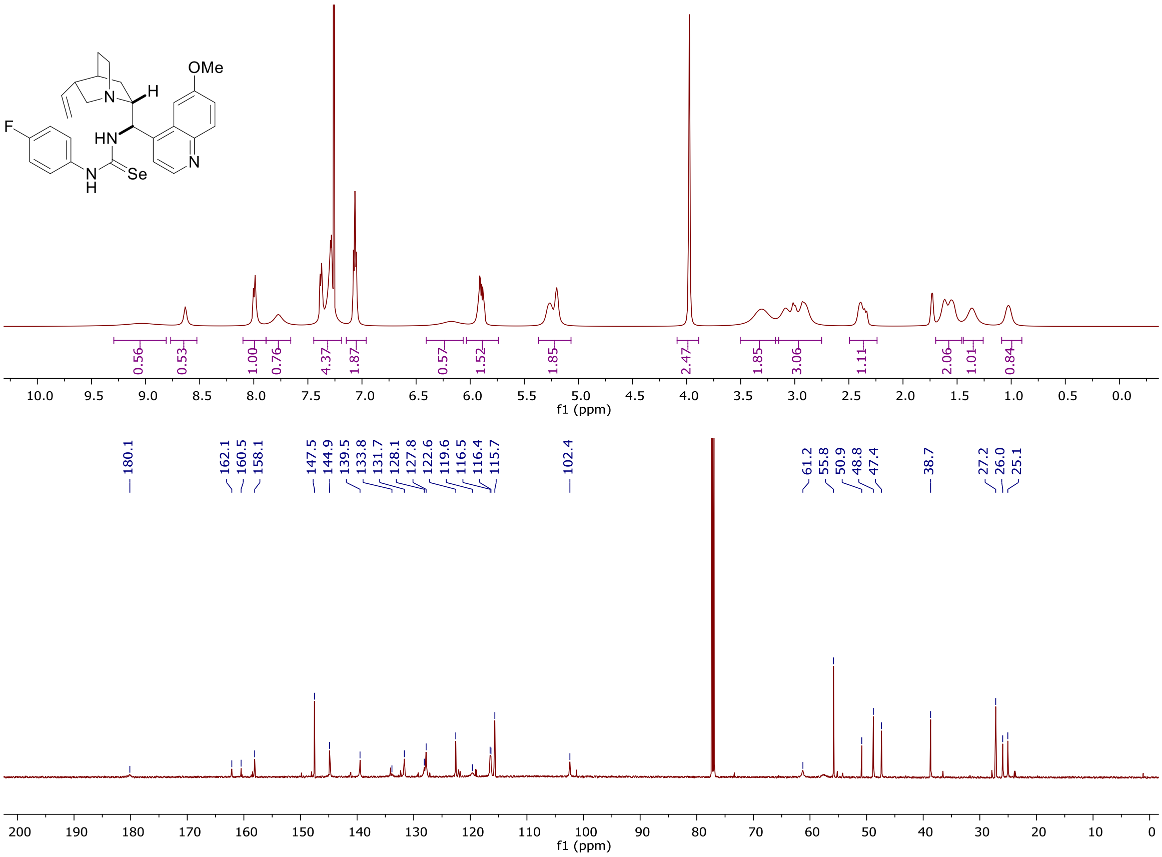

Figure S11. 1H and 13C NMR spectra of *N*-[4-fluorophenyl]-*N*′-[(8*R*,9*R*)-6′-methoxycinchonan-9-yl]selenourea *e*QD-**7f**.

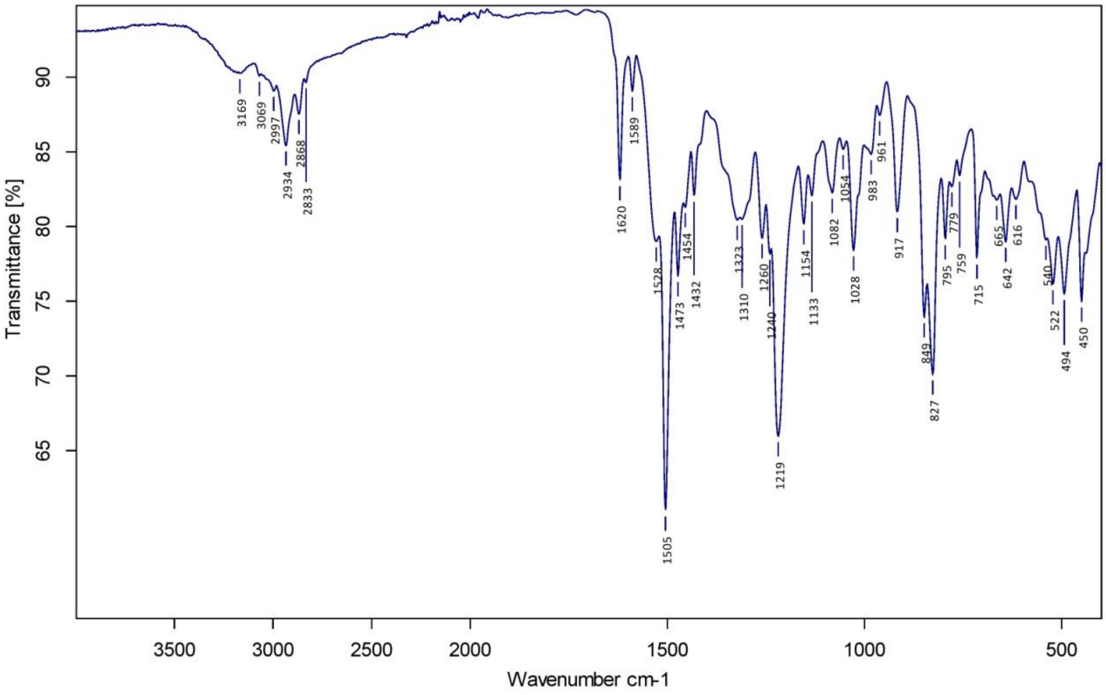

Figure S12. IR spectra of *N*-[4-fluorophenyl]-*N'*-[(8*R*,9*R*)-6'-methoxycinchonan-9-yl]selenourea *e*QD-**7f**.

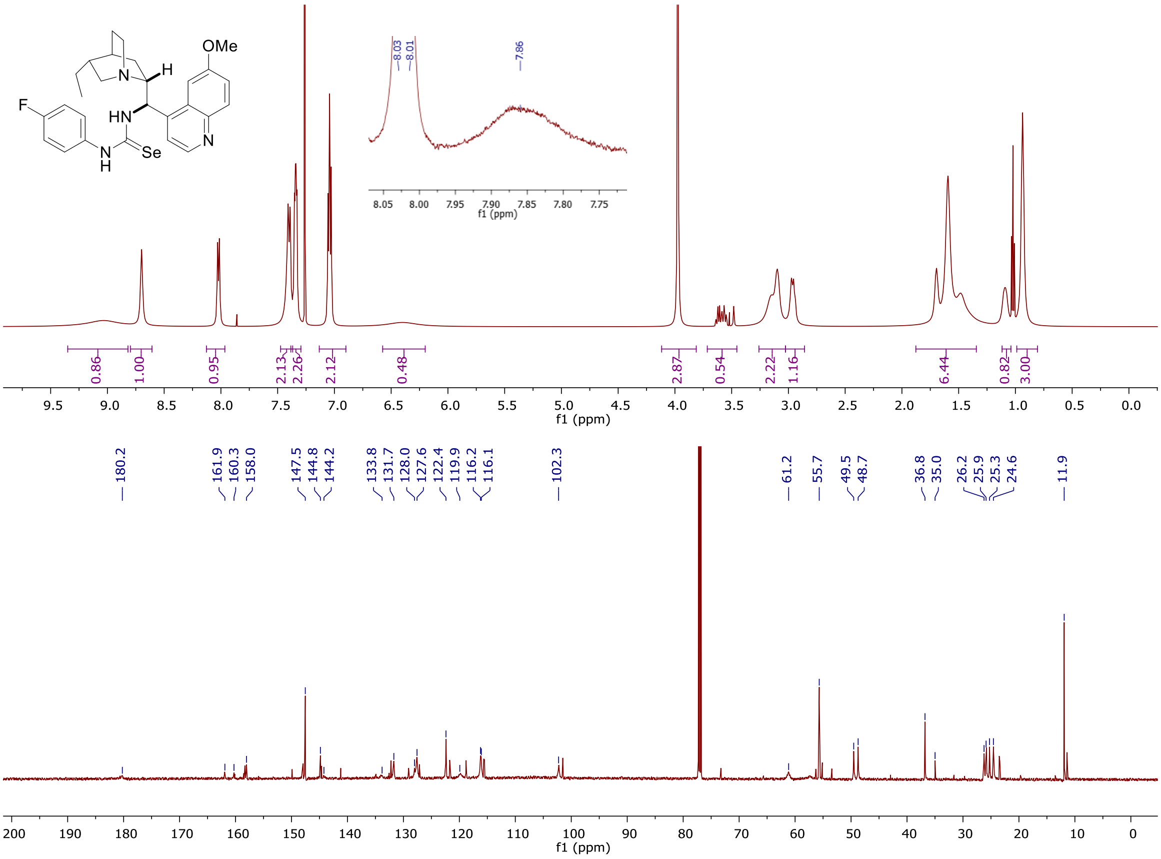

Figure S13. 1H and 13C NMR spectra of *N*-[4-fluorophenyl]-*N'*-[(8*R*,9*R*)-10,11-dihydro-6'- methoxycinchonan-9-yl]selenourea *e*DHQD-**7g**.

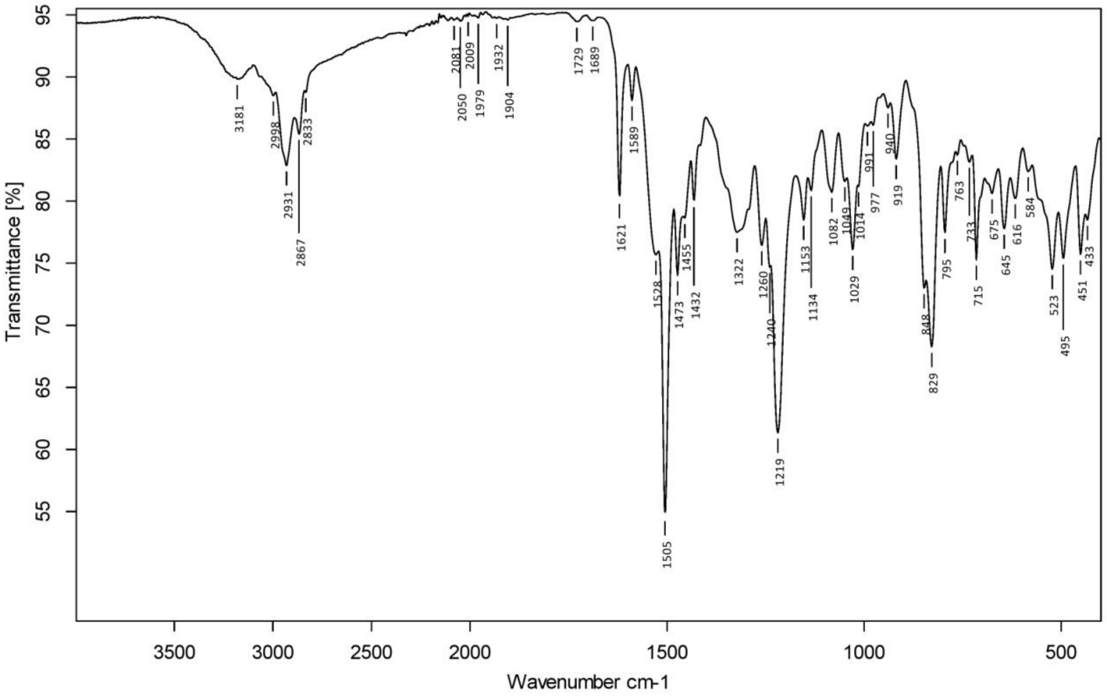

Figure S14. IR spectra of *N*-[4-fluorophenyl]-*N*′-[(8*R*,9*R*)-10,11-dihydro-6′-methoxycinchonan-9-yl]selenourea *e*DHQD-**7g**.

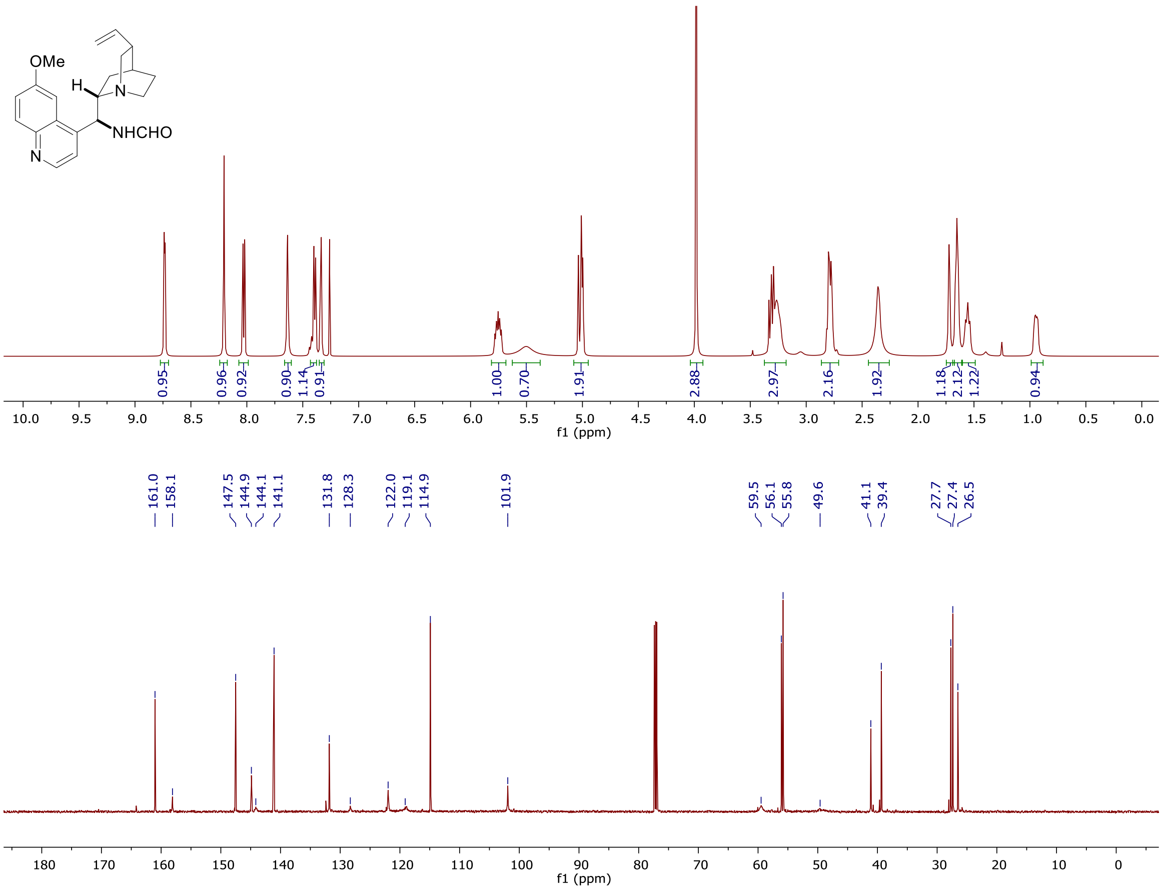

Figure S15. 1H and 13C NMR spectra of *N*-[(8*S*,9*S*)-6′-methoxycinchonan-9-yl]formamide **8a.**

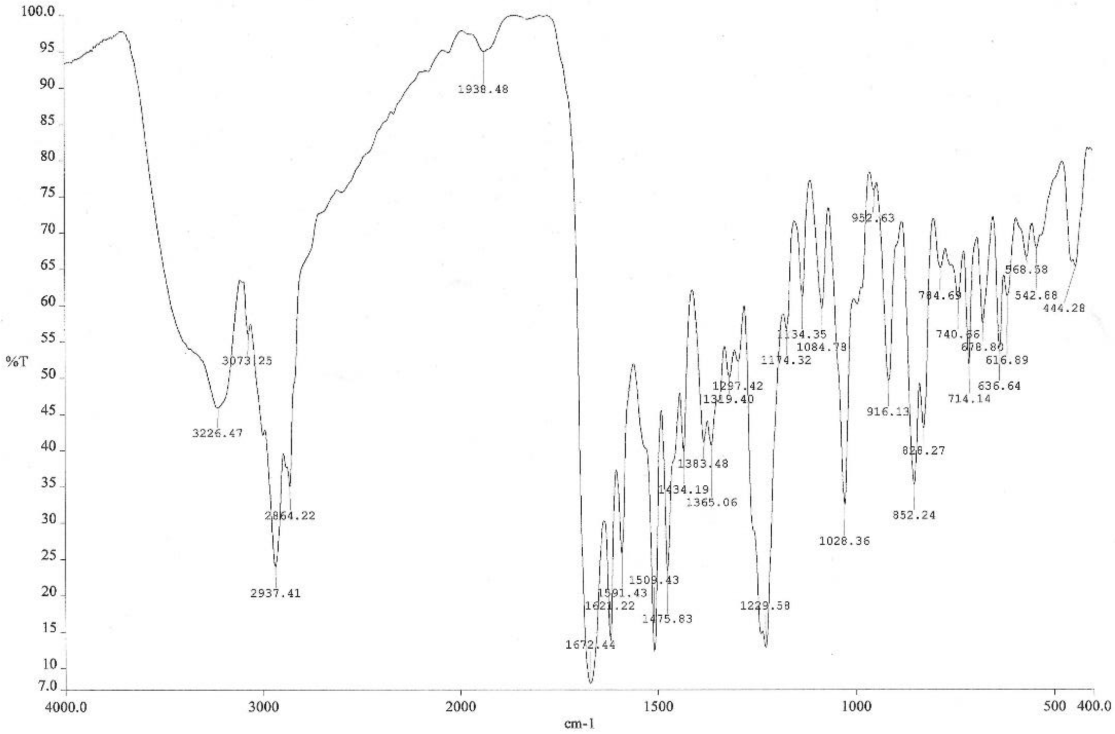

Figure S16. IR spectra of *N*-[(8*S*,9*S*)-6′-methoxycinchonan-9-yl]formamide **8a.**

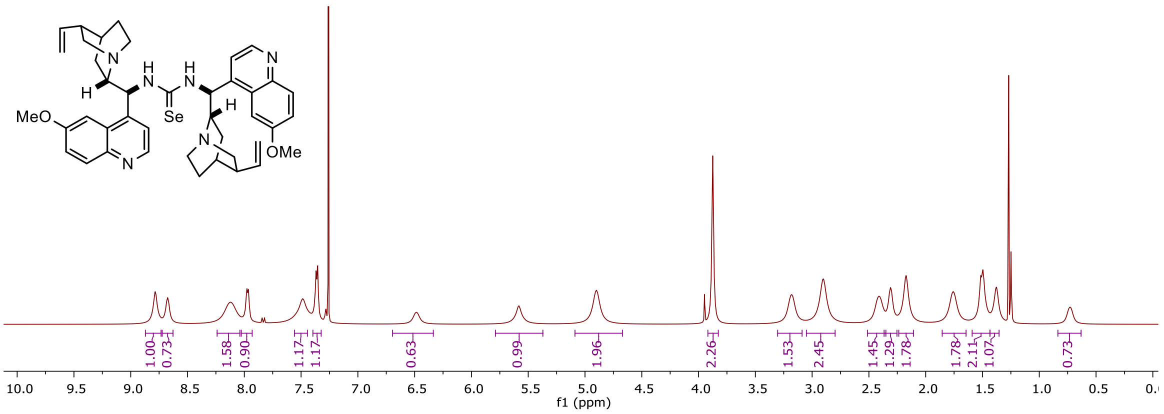

Figure S17. 1H NMR spectra of *N,N′*-bis[(8*S*,9*S*)-6′-metoxycinchonan-9-yl]selenourea **10a.**

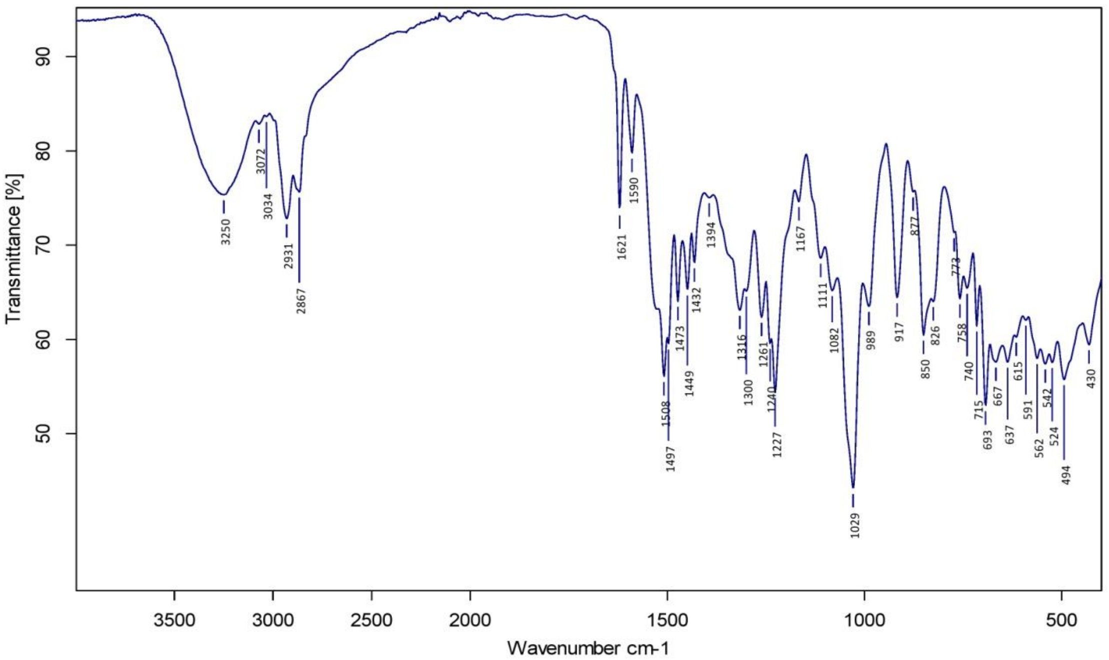

Figure S18. IR spectra of *N,N′*-bis[(8*S*,9*S*)-6′-metoxycinchonan-9-yl]selenourea **10a.**

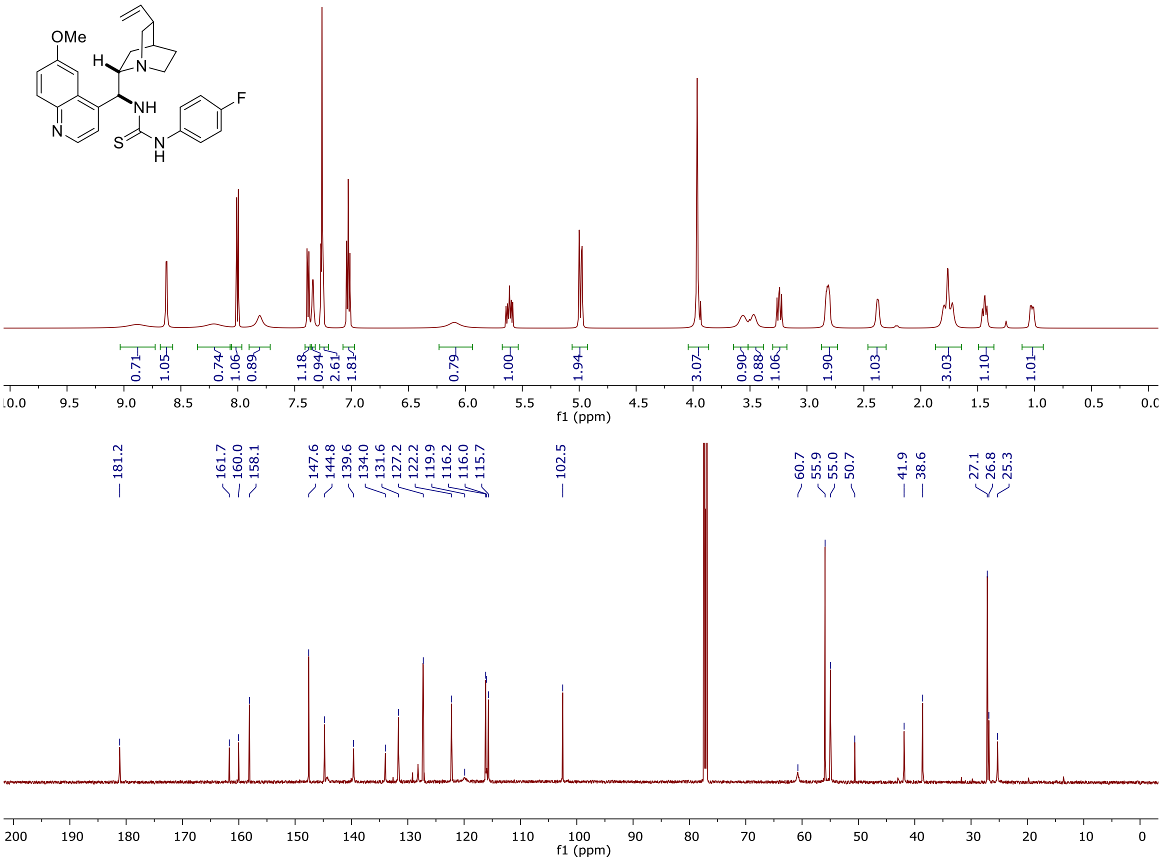

Figure S19. 1H and 13C NMR spectra of *N*-[4-fluorophenyl]-*N′*-[(8*S*,9*S*)-6′-methoxycinchonan-9- yl]thiourea *e*QN-**12a**.

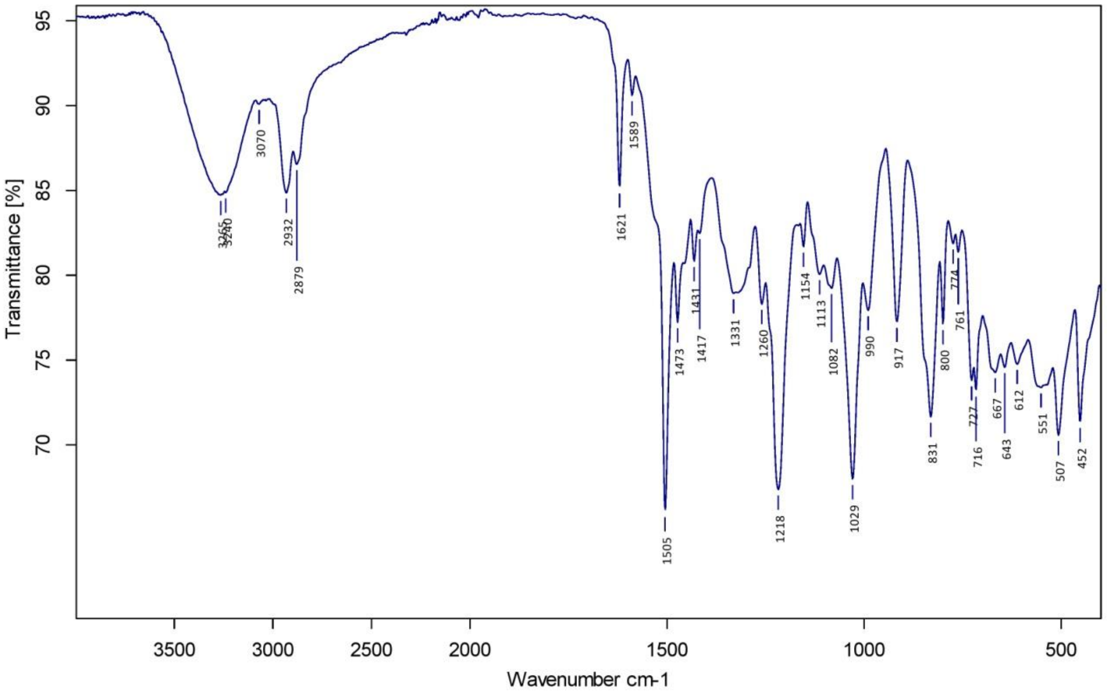

Figure S20. IR spectra of *N*-[4-fluorophenyl]-*N'*-[(8*S*,9*S*)-6'-methoxycinchonan-9-yl]thiourea *e*QN-**12a**.

2. HPLC data

2.1. Michael addition of nitromethane to trans-chalcone

(Chiralcel AD-H column, hexane/*i*-PrOH 9:1, flow rate 1.0 mL/min, λ = 254 nm)

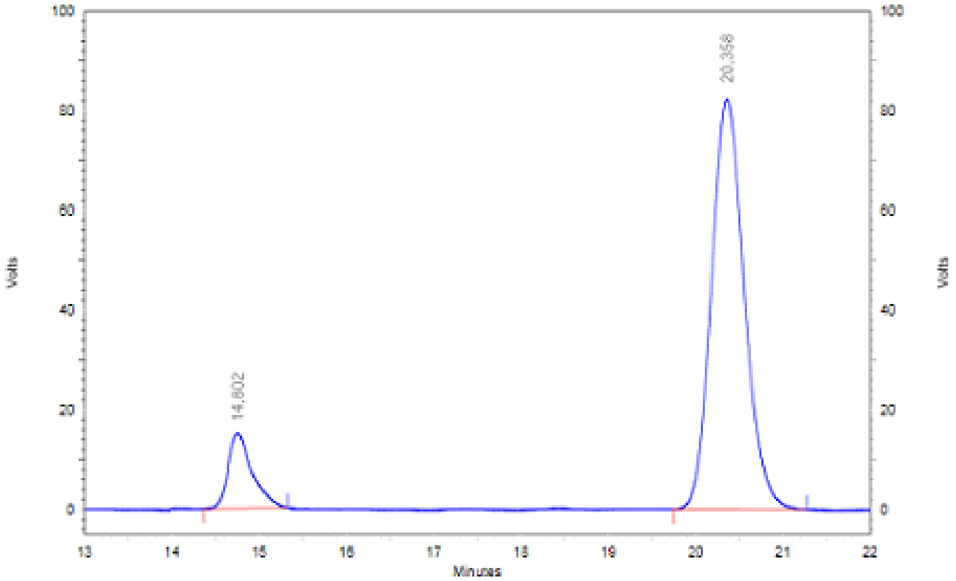

Retention Time	Area	Area %	Height	Height %
14,802	219734	9,27	18011	17,95
20,358	2150647	90,73	82316	82,05
Totals	2370381	100,00	100327	100,00

Figure S21. HPLC chromatogram for **14:** sample obtained with catalyst *e*QN-**7a** (81% ee).

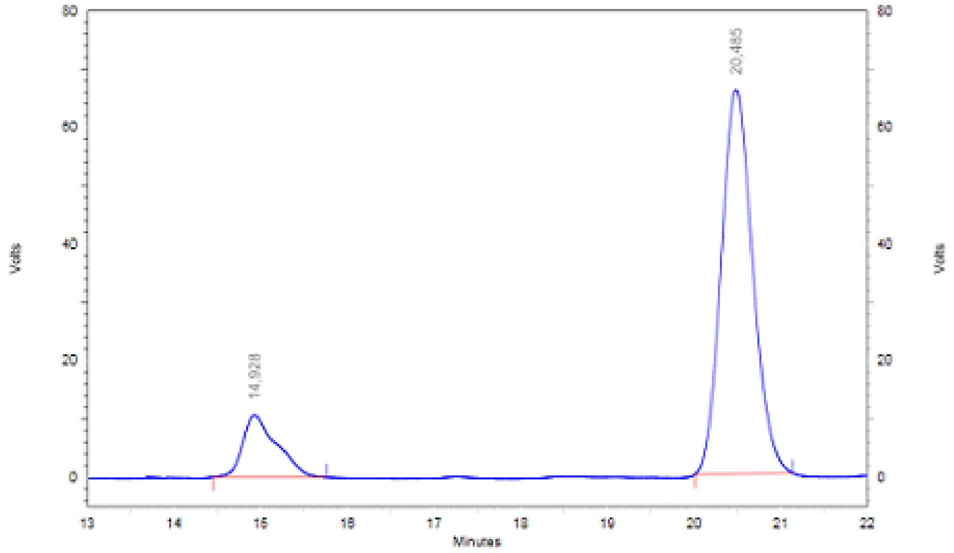

Retention Time	Area	Area %	Height	Height %
14,928	290990	14,92	10678	13,96
20,485	1659479	85,08	65824	86,04
Totals	1950469	100,00	76502	100,00

Figure S22. HPLC chromatogram for **14**: sample obtained with catalyst *e*QN-**7b** (70% ee).

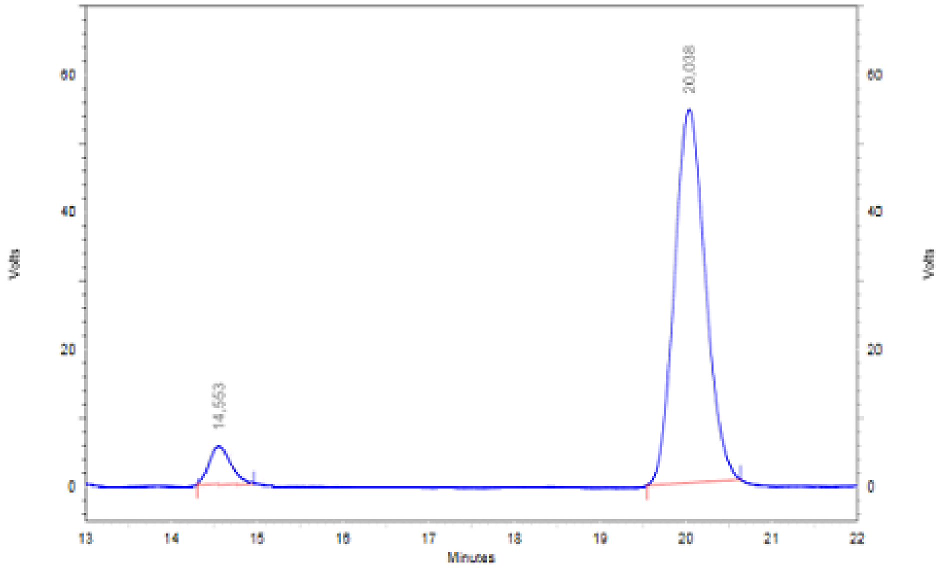

Retention Time	Area	Area %	Height	Height %
14,553	35947	2,58	3203	5,56
20,038	1359921	97,42	54389	94,44
Totals	1395868	100,00	57592	100,00

Figure S23. HPLC chromatogram for **14**: sample obtained with catalyst *e*QN-**7c** (95% ee).

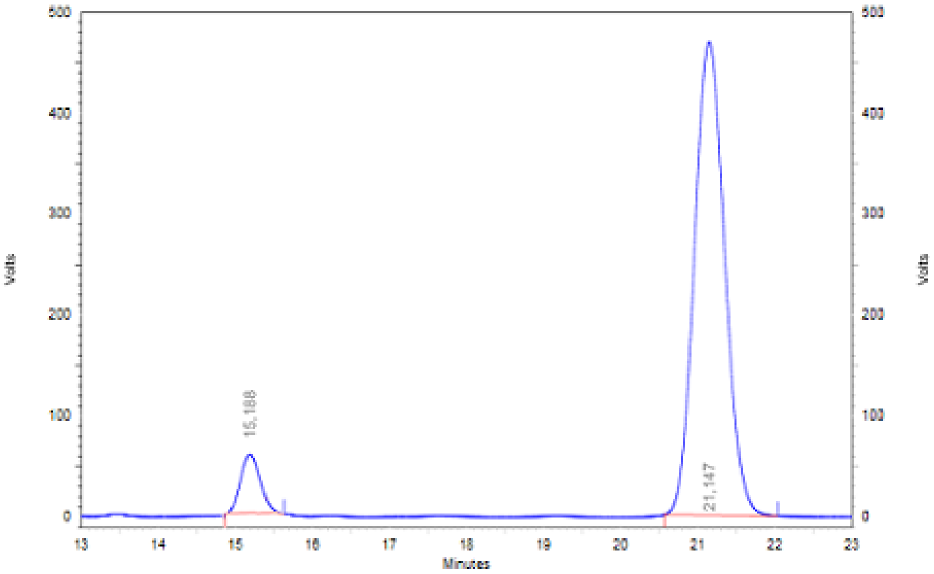

Retention Time	Area	Area %	Height	Height %
15,188	619388	4,62	44635	8,69
21,147	12779893	95,38	468927	91,31
Totals	13399281	100,00	513562	100,00

Figure S24. HPLC chromatogram for **14**: sample obtained with catalyst *e*CD-**7d** (91% ee).

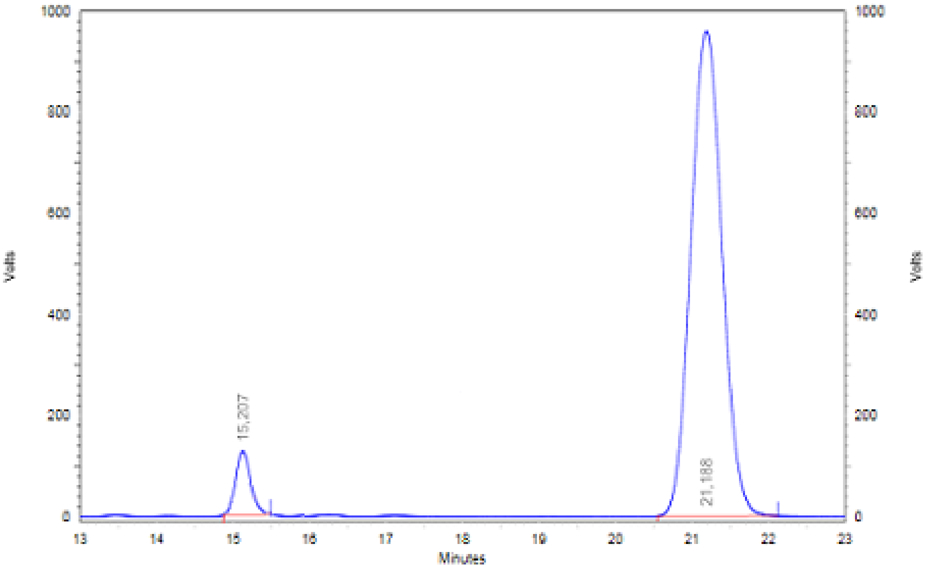

Retention Time	Area	Area %	Height	Height %
15,207	692689	2,48	178203	15,68
21,188	27238328	97,52	957962	84,32
Totals	27931017	100,00	1136165	100,00

Figure S25. HPLC chromatogram for **14**: sample obtained with catalyst *e*DHQN-**7e** (95% ee).

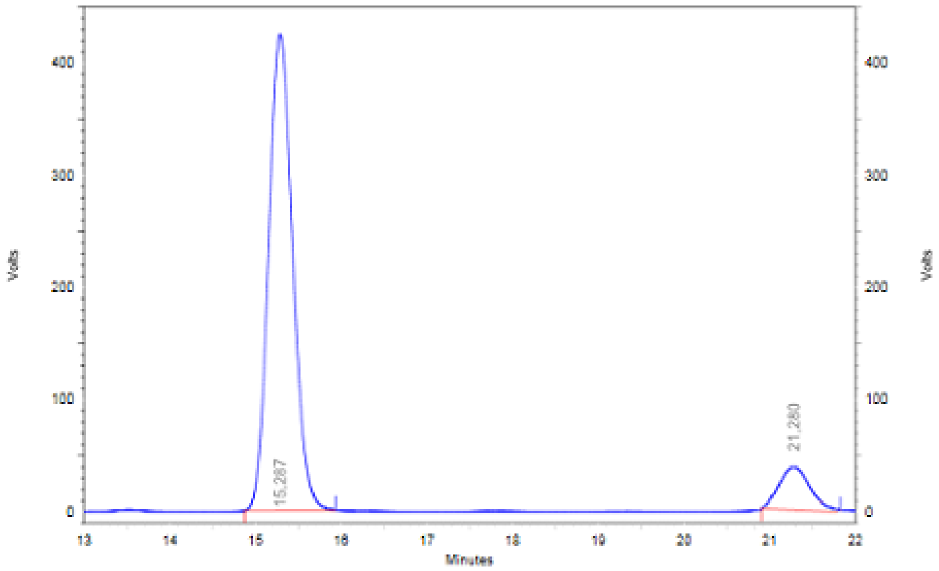

Retention Time	Area	Area %	Height	Height %
15,287	8275168	93,26	424402	93,40
21,280	597842	6,74	30003	6,60
Totals	8873010	100,00	454405	100,00

Figure S26. HPLC chromatogram for **14**: sample obtained with catalyst *e*QD-**7f** (87% ee).

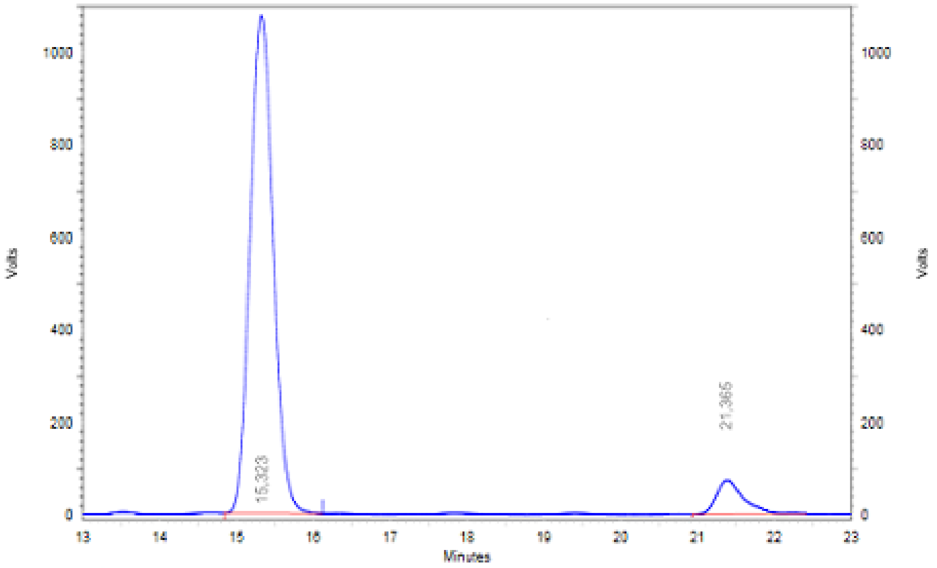

Retention Time	Area	Area %	Height	Height %
15,323	22494720	93,22	1076675	89,27
21,365	1636067	6,78	129431	10,73
Totals	24130787	100,00	1206106	100,00

Figure S27. HPLC chromatogram for **14**: sample obtained with catalyst eDHQD-**7g** (86% ee).

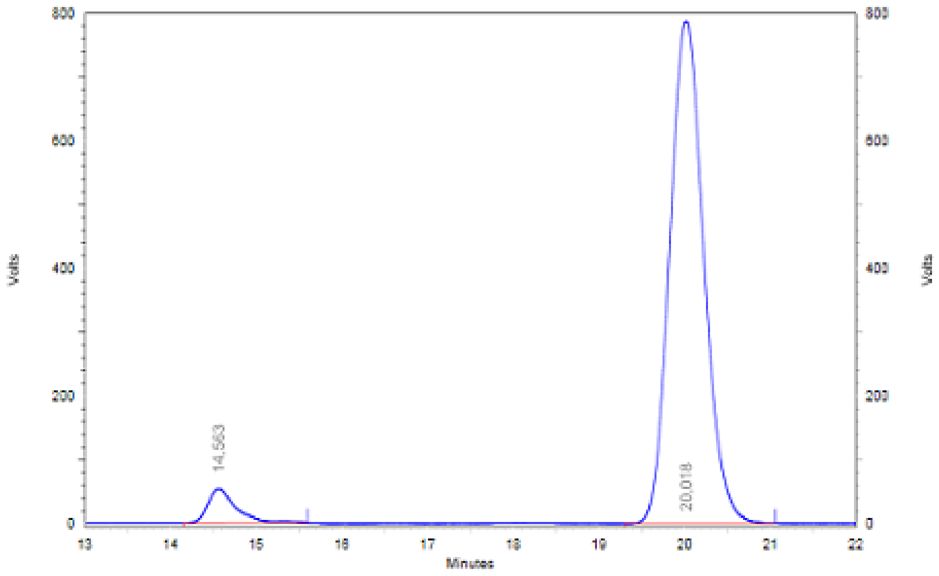

Retention Time	Area	Area %	Height	Height %
14,563	1223538	5,34	53870	6,41
20,018	21694987	94,66	786695	93,59
Totals	22918525	100,00	840565	100,00

Figure S28. HPLC chromatogram for **14**: sample obtained with catalyst *e*QN-**12a** (89% ee).

Data File: C:\Documents and Settings\SPECTRA\Pulpit\MZB\MBK47.dat
Method: C:\ChromQuest\Enterprise\Projects\Default\Method\RK-MD-1.met
Printed: 2021-01-15 11:56:26
ADH_90_10_254nm_1ml_min

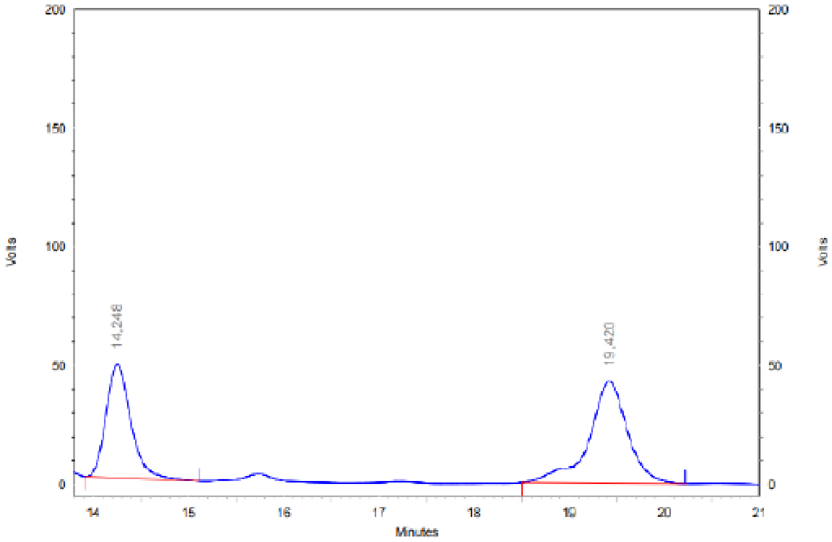

Peak#	Ret. Time	Area	Area %	Height	Height %
1	14.248	1027583	49.38	47709	52.78
2	19.420	1053468	50.62	42689	47.22
Total		2081051	100.00	90398	100.00

Figure S29. HPLC chromatogram for racemic **14** without catalyst **7**.

2.2. Sulfa-Michael addition of thioacetic acid to trans-chalcone

(Chiralcel AS-H column, hexane/*i*-PrOH 9:1, flow rate 1.0 mL/min, λ = 254 nm)

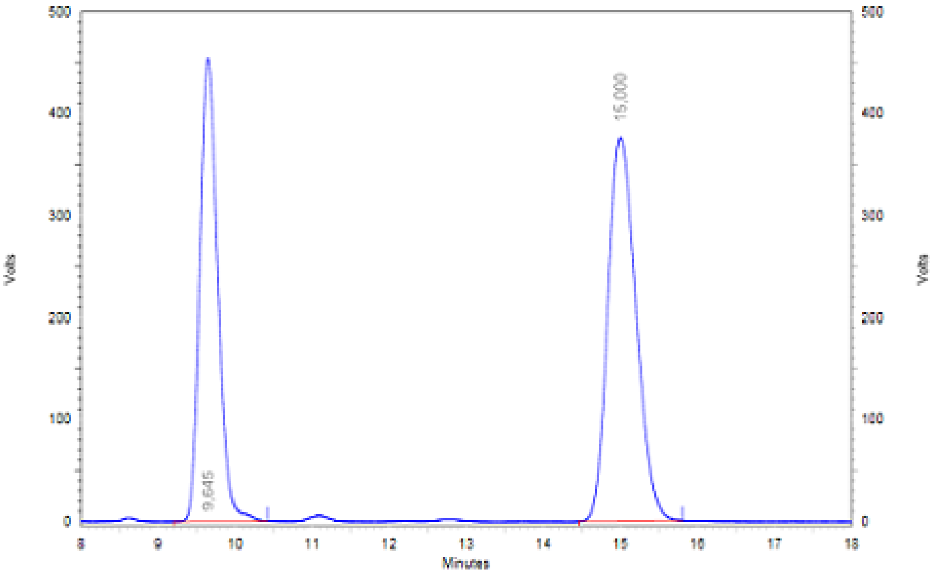

Retention Time	Area	Area %	Height	Height %
9,645	7398964	43,63	454245	54,69
15,000	9559617	56,37	376303	45,31
Totals	16958581	100,00	830548	100,00

Figure S30. HPLC chromatogram for **15:** sample obtained with catalyst *e*QN-**7a** (13% ee).

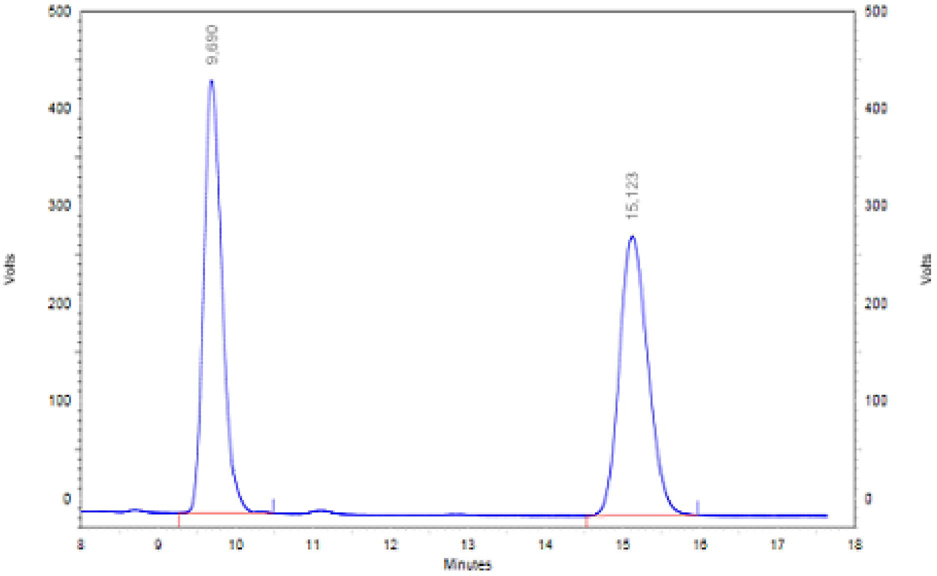

Retention Time	Area	Area %	Height	Height %
9,690	7294361	48,48	443991	60,84
15,123	7750920	51,52	285835	39,16
Totals	15045281	100,00	729826	100,00

Figure S31. HPLC chromatogram for **15:** sample obtained with catalyst *e*QN-**7b** (3% ee).

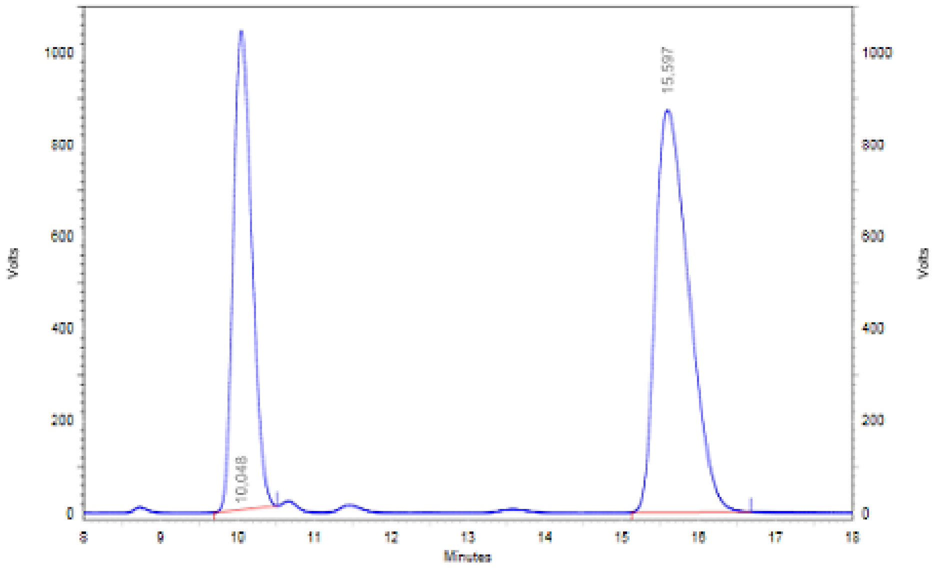

Retention Time	Area	Area %	Height	Height %
10,048	17443372	39,72	1040394	54,36
15,597	26473383	60,28	873530	45,64
Totals	43916755	100,00	1913924	100,00

Figure S32. HPLC chromatogram for **15:** sample obtained with catalyst *e*QN-**7c** (21% ee).

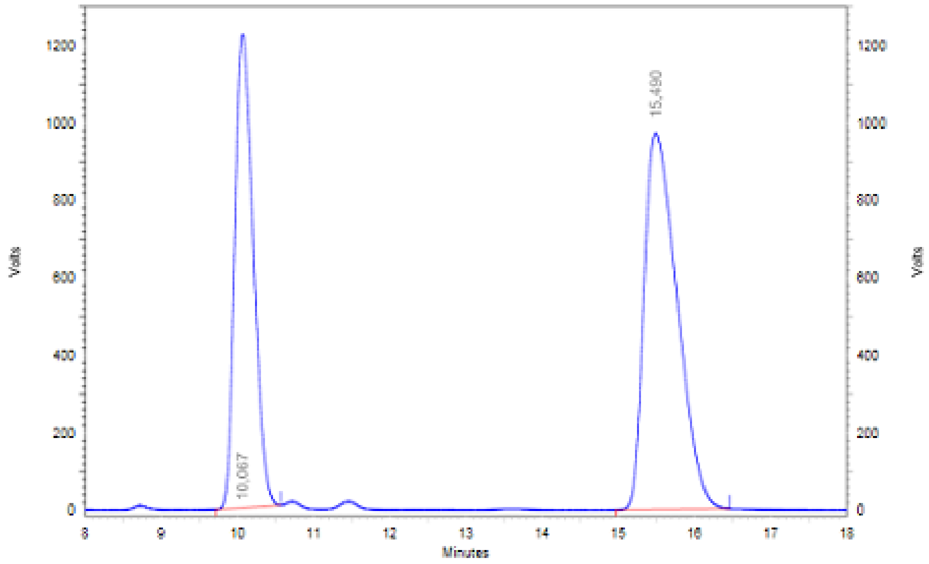

Retention Time	Area	Area %	Height	Height %
10,067	20722516	41,44	1223857	55,72
15,490	29279474	58,56	972585	44,28
Totals	50001990	100,00	2196442	100,00

Figure S33. HPLC chromatogram for **15:** sample obtained with catalyst *e*CD-**7d** (17% ee).

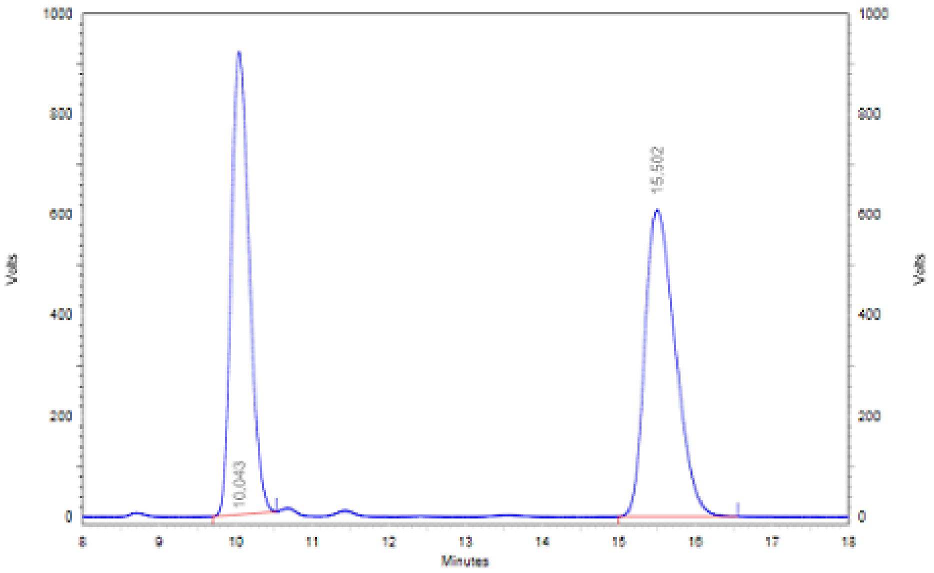

Retention Time	Area	Area %	Height	Height %
10,043	10852273	39,23	918530	60,12
15,502	16810927	60,77	609242	39,88
Totals	27663200	100,00	1527772	100,00

Figure S34. HPLC chromatogram for **15:** sample obtained with catalyst *e*DHQN-**7e** (21% ee).

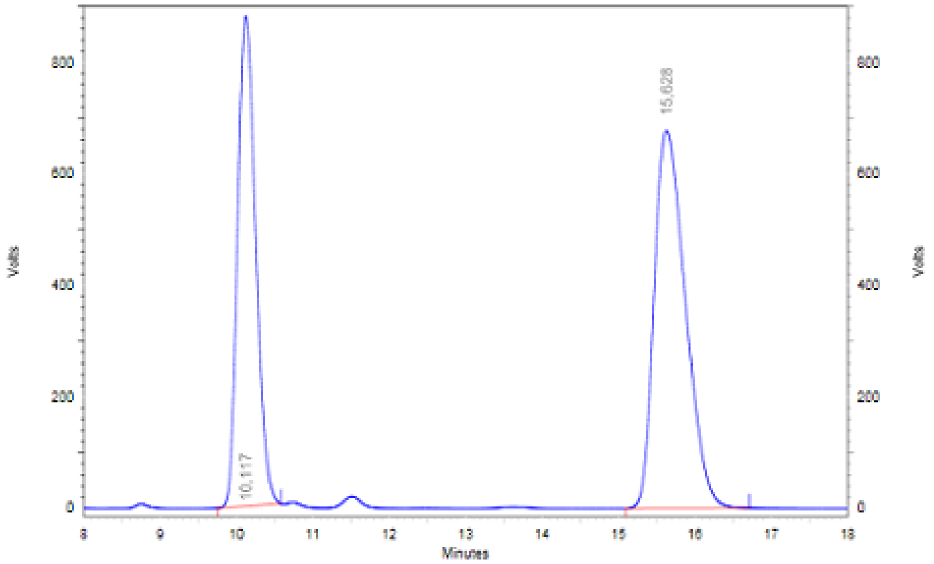

Retention Time	Area	Area %	Height	Height %
10,117	14384230	42,83	878691	56,50
15,628	19202372	57,17	676598	43,50
Totals	33586602	100,00	1555289	100,00

Figure S35. HPLC chromatogram for **15:** sample obtained with catalyst *e*QD-**7f** (14% ee).

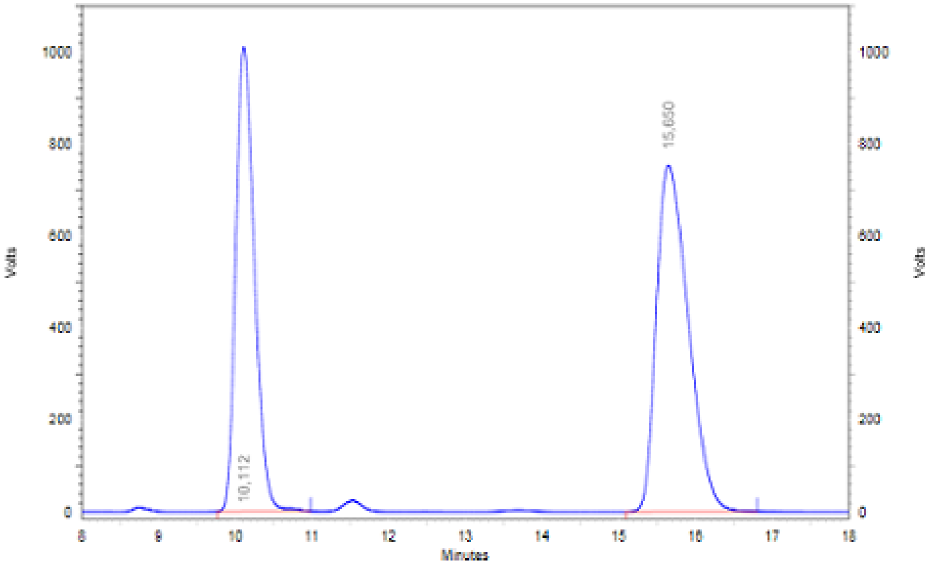

Retention Time	Area	Area %	Height	Height %
10,112	17085326	43,94	1008589	57,23
15,650	21795086	56,06	753786	42,77
Totals	38880412	100,00	1762375	100,00

Figure S36. HPLC chromatogram for **15:** sample obtained with catalyst *e*DHQD-**7g** (12% ee).

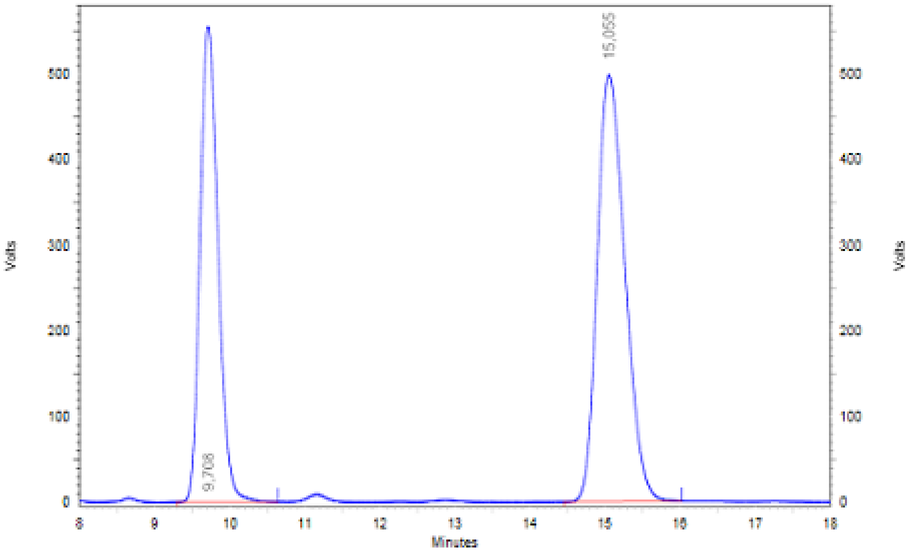

Retention Time	Area	Area %	Height	Height %
9,708	9139615	41,65	555335	52,72
15,055	12805305	58,35	498067	47,28
Totals	21944920	100,00	1053402	100,00

Figure S37. HPLC chromatogram for **15:** sample obtained with catalyst *e*QN-**12a** (17% ee).

Data File: C:\Documents and Settings\SPECTRA\Pulpit\MZB\MZB_737_ASH_90_10_254_1_CR.dat
Method: C:\ChromQuest\Enterprise\Projects\Default\Method\RK von AAchen\DO_testow_9do1_default.met
Printed: 2021-01-15 13:52:35
ASH_90_10_254nm_1ml_min

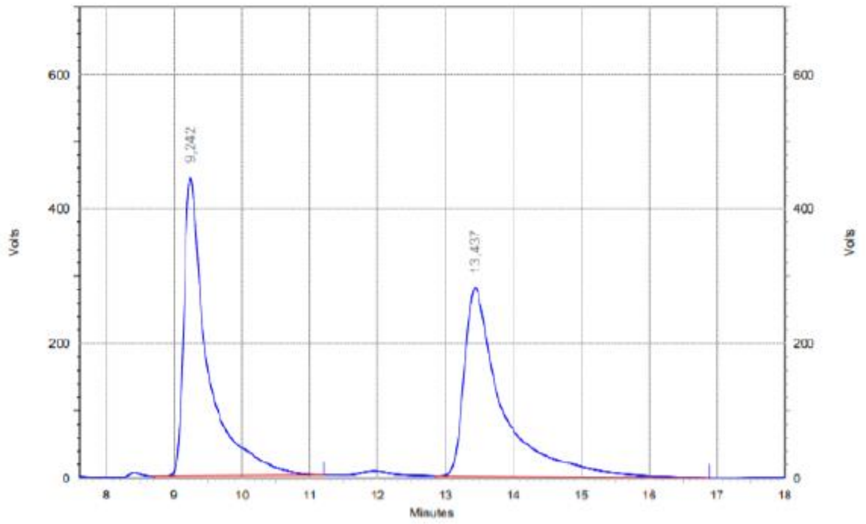

Peak#	Ret. Time	Area	Area %	Height	Height %
1	9.242	10717047	49.85	442263	61.34
2	13.437	10780306	50.15	278703	38.66
Total		21497353	100.00	720966	100.00

Figure S38. HPLC chromatogram for racemic **15** without catalyst **7**.
